# Electric Eel foraging optimization based control design of islanded microgrid

**DOI:** 10.1038/s41598-025-91006-y

**Published:** 2025-03-09

**Authors:** M. A. Ebrahim, Ahmed S. Ragab, Beshoy Abdou Aziz, H. A. AbdelHadi

**Affiliations:** 1 Electrical Engineering Department, Faculty of Engineering at Shoubra, Benha University, Cairo, Egypt; 2 Electrical Engineering Department, Modern Academy for Engineering and Technology, Cairo, Egypt; 3 Electrical Engineering Department, Benha Faculty of Engineering, Benha University, Qalubia, Egypt

**Keywords:** Microgrid, Hierarchical control, Synchronization, Optimization, HIL, Electrical and electronic engineering, Energy grids and networks, Power distribution

## Abstract

Designing control systems for islanded microgrids poses significant challenges due to the absence of inertia and parameter uncertainties. These factors increase the complexity of traditional methods when applied to highly nonlinear and interdependent systems. To address this issue, a novel Electric Eel Foraging Optimization (EEFO) technique is proposed for tuning control parameters within a hierarchical structure of primary and secondary control levels. The control system employs proportional resonant (PR) controllers for voltage and current regulation, alongside a synchronization loop to enable seamless grid reconnection. Comparative analysis with Particle Swarm Optimization (PSO) and Grey Wolf Optimization (GWO) confirms EEFO’s superior convergence speed and solution quality performance. Simulation results using MATLAB/SIMULINK demonstrate effective active power sharing, minimal overshoot and settling times for voltage (2.4%, 0.25 s) and frequency (0.42%, 0.53 s), and seamless grid reconnection. Experimental validation using hardware-in-the-loop (HIL) real-time emulation further verifies the feasibility and robustness of the proposed approach for practical microgrid applications.

## Introduction

The rising concerns about the environment and energy costs force the power sector to undergo significant transformation. As a result, renewable energy-based distributed generation (DG) is considered an effective solution to this issue. These DGs are integrated with energy storage devices and loads to form a controlled microgrid that can operate connected to the utility grid or as a standalone or islanded system^1^.

Power converters are crucial components in microgrids, facilitating the efficient processing and integration of energy generated by renewable energy sources. Power converters can be divided into two types based on how they operate in an alternating current microgrid: grid-feeding and grid-forming^2^. Grid-feeding power converters are primarily designed to provide power to an electrified grid. They can be considered as an ideal current source in parallel with a high impedance. On the other hand, the grid-forming converters set the microgrid’s frequency and voltage amplitude by using a proper control loop. They can be modeled as an ideal AC voltage source in series with a low output impedance.

Grid-feeding power converters rely on the presence of a grid-forming unit or a local synchronous generator to establish the voltage amplitude and frequency of the AC microgrid, making them unsuitable for independent operation in island mode. Grid-feeding converters are typically managed by high-level controllers, such as power plant controllers or maximum power point tracking (MPPT) controllers, which define the reference values for active and reactive power. These converters are suitable for controlling renewable energy sources like photovoltaic (PV) and wind^3^.

Grid-forming units generally employ a three-level hierarchical control structure: primary, secondary, and tertiary controls. The primary control is responsible for maintaining stable voltage and frequency operation while ensuring accurate power sharing among inverter units^4,5^. However, the primary control results in deviations from nominal values in frequency and voltage^6^. These deviations are eliminated by the secondary control^7^. A synchronization control could be added to the secondary control to ensure a seamless transition from islanded to grid-connected mode. Tertiary control ensures the optimal operation of the microgrid by managing power flow between the microgrid and the utility grid, taking into account both economic and environmental considerations^8^. The tertiary control is not addressed in this paper. In industrial applications, grid-forming power converters are often powered by a stable DC voltage source such as a battery, a fuel cell (FC), or another primary source^9^.

**Fig. 1:**
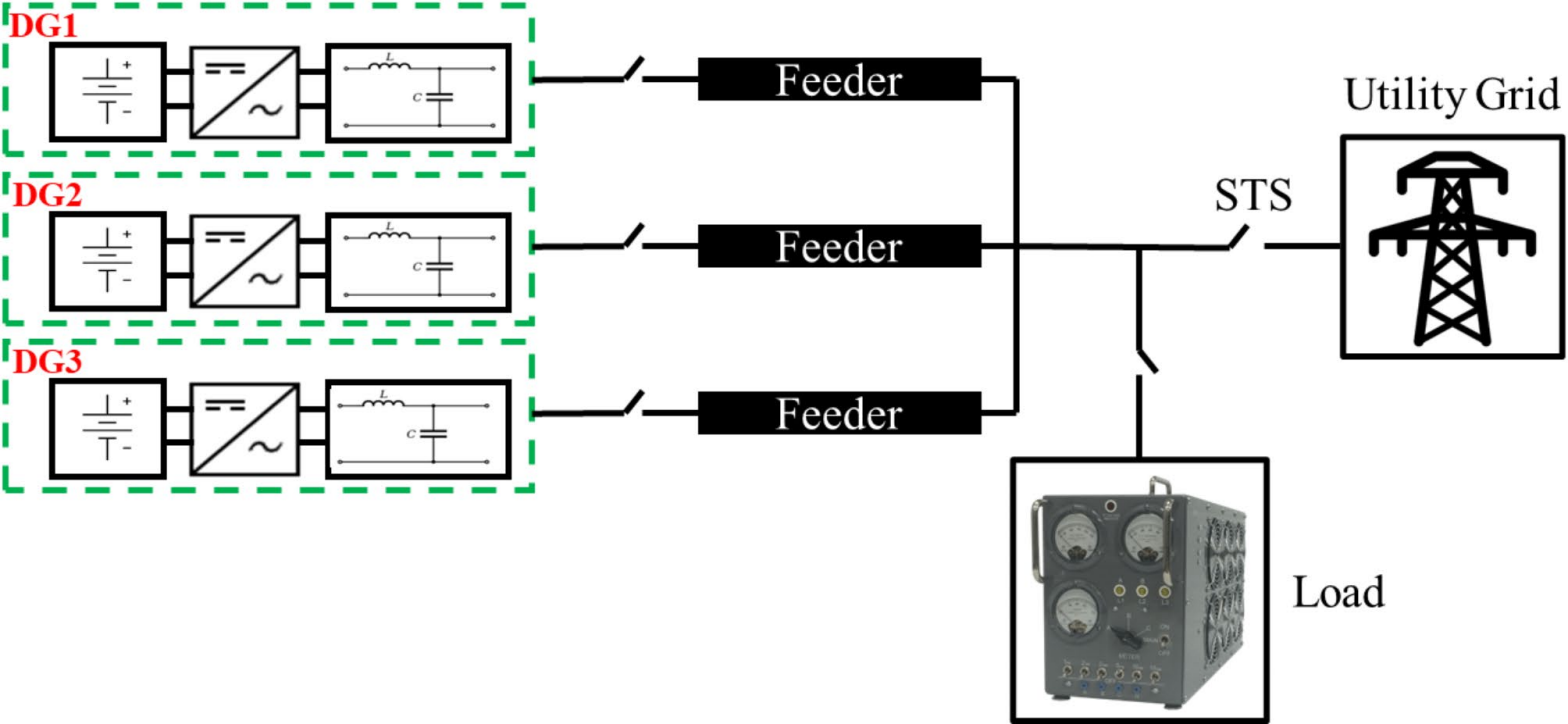
Microgrid structure under study.

Maintaining a stable and reliable microgrid despite parameter uncertainty is vital for proper operation. Because of a lack of inertia and poor selection of controller parameters, the microgrid could suffer from severe variations in voltage and frequency especially when operating in islanded mode. Therefore, careful controller design is essential to ensure the required power quality and control performance under different operating conditions. Classic methods for tuning individual controllers include heuristic approaches such as frequency domain methods that account for gain and phase margins, Ziegler Nichols method, and analytical methods such as pole placement. However, there are several controllers in a microgrid system and the result of tuning one control parameter is likely to affect other parameters tuning. So, using these methods to tune multiple DG controllers would be rather complicated and inefficient. Recently, Numerous metaheuristic optimization techniques have been proposed to help solve a variety of engineering issues, including the design of control systems^10^. These techniques would be the best option since there is a lot of flexibility in defining fitness functions (FF) which express control objectives, and optimal parameters can be obtained using automatic computerized iterative searches.

Regarding the topic of microgrids, these meta-heuristic methods have been applied in the literature to optimize the design of controllers for improving power quality and dynamic performance. Most research in this domain concentrates on enhancing the performance of proportional-integral (PI) controllers in microgrids^11–17^. However, literature indicates that PR controllers outperform PI controllers in microgrid applications, particularly in terms of voltage and frequency regulation, power quality, and power-sharing^18–21^. Unlike the conventional PI control strategy, which operates in the $\:dq$ rotating synchronous frame, PR controllers work in the $\:\alpha\:\beta\:$ stationary frame, offering superior sinusoidal reference tracking without steady-state errors and enhanced disturbance rejection capabilities. Additionally, PR controllers can be paired with harmonic compensators to effectively suppress selective positive and negative harmonics. Also, this approach eliminates the need for feedforward elements and decoupling terms, simplifying the control design. Given these advantages, PR controllers are employed in this study.

Despite their benefits, research on the optimization of PR-based microgrids developed in αβ stationary reference frame, especially in islanded mode, remains limited. Moreover, the optimization of the transition process from islanded to grid-connected mode has not been thoroughly explored. To address these gaps, this paper focuses on optimizing the performance of PR-based islanded microgrids and considers the optimization of synchronization control loops for a seamless transition into grid-connected mode. A novel optimization strategy, Electric Eel Foraging Optimization (EEFO), is proposed for tuning control parameters. The optimization process is directly applied to the nonlinear model, incorporating the fitness function into the simulation results. This methodology produces more practical and accurate outcomes compared to traditional tuning approaches, which are typically based on linearized models. The effectiveness of the proposed strategy is validated through comparisons with two well-established optimization techniques, PSO and GWO.

The microgrid is comprised of three parallel DG units feeding a common load as indicated in.

Figure 1. One unit operates as a grid-feeding unit while the other two operate as grid-forming units to ensure the continuity of operation in case of failure of one unit thus increasing the reliability of the system. Each DG includes a three-phase two-level power converter, and LC filter, as well as an ideal DC source representing the DC link of a typical renewable energy generation system or energy storage. The microgrid could be connected to the utility grid via a static transfer switch (STS).

The microgrid model was developed and simulated using MATLAB/SIMULINK (version R2021a)^22^. Additionally, the proposed system was experimentally validated using a hardware-in-the-loop (HIL) real-time emulation setup, implemented with the C2000 Microcontroller LaunchPad XL-TMS320F28379D kit.

This paper’s main contributions can be highlighted as follows:

- Application of a novel optimization technique, EEFO, for optimal design of control parameters in PR-based islanded microgrid modeled in $\:\alpha\:\beta\:$ stationary reference frame. The optimization framework also addresses synchronization control to facilitate a seamless transition from islanded into grid-connected operation.
- The proposed optimization approach demonstrates robustness and superior performance, particularly in terms of convergence speed and solution quality, when compared to established techniques such as PSO and GWO.
- The effectiveness of the optimized controllers is experimentally validated through HIL emulation, highlighting their potential for practical deployment in real-world microgrid applications.

The organization of the paper is as follows: Section II introduces the main ideas behind Electric Eel Foraging Optimization (EEFO). Sections III through VI describe the primary and secondary control levels, as well as their optimal parameter tuning methodology. The simulation results and experimental validation are detailed in Sections VII and VIII, respectively. Section IX provides the conclusion of the study, while Section X outlines potential directions for future research.

## Electric Eel foraging optimization

Electric Eel Foraging Optimization (EEFO) is a recently proposed swarm-based optimization technique inspired by the intelligent group foraging behaviors of electric eels found in nature^23^. Electric eels are well-known for their powerful discharge capabilities in freshwater fish. Eels generate electricity using three pairs of electric organs which contain thousands of electricity generation cells known as electrocytes. These electrocytes can store power in the same way as batteries do. Because of their poor eyesight, eels use low electric discharge to communicate with each other and to track and locate prey. On the other hand, they use high electric discharge for hunting and surviving enemies. To pursue prey and engage in social predation, eels coordinate actions including interacting, resting, hunting, and migrating. These actions are described in the following points:

- The interaction shows that each eel engages cooperatively with other individuals based on their positions. The interactive behavior allows electric eels to move to different positions in the search space, which can greatly aid in the exploration of EEFO throughout the search space.
- Before electric eels engage in resting behavior in EEFO, the resting area must be established. When the resting area is determined, eels will move to it to rest. The resting behavior enhances exploitation as iterations proceed.
- When eels go hunting, they communicate with each other forming an electrified circle around the prey. This electrified circle becomes the hunting area where eels stun their prey with a killing high-voltage current. An eel can detect a prey’s location through low electric discharges.
- Migration behavior mimics the movement of the eel from the resting area to the hunting area when prey is found.

**Fig. 2:**
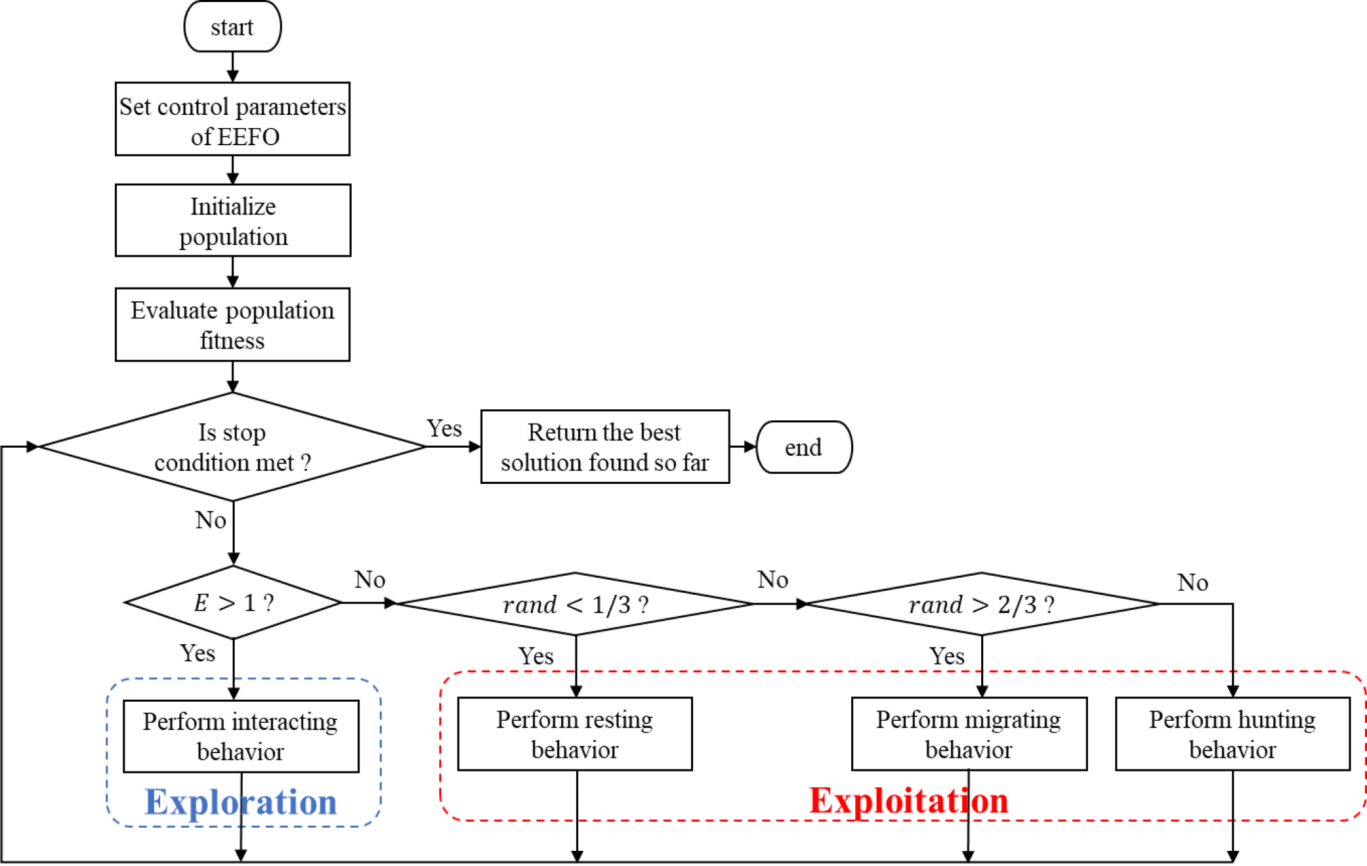
EEFO Flowchart.

In EEFO, an energy factor (E) is defined which determines the searching behavior and manages the balance between exploration and exploitation. Interacting behavior performs global search resulting in exploration while resting, migrating, and hunting contribute more to exploitation. Figure 2presents the flowchart of the EEFO optimization algorithm. The complete mathematical modeling and more details can be found in the original article on the algorithm^23^.

## Control of grid-feeding converter

**Fig. 3:**
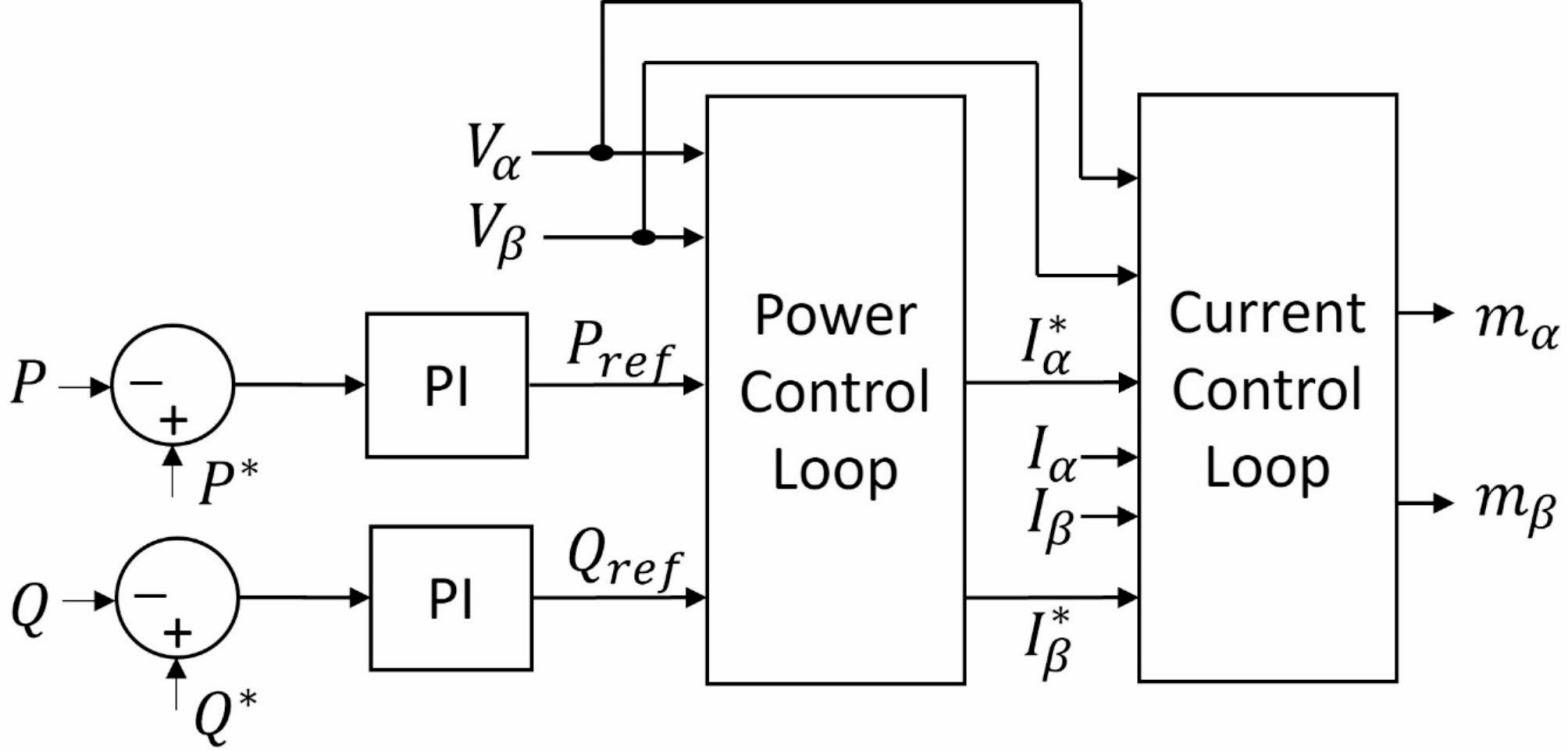
Control block diagram of grid-feeding converter.

Figure 3 illustrates the control block diagram of the grid-feeding converter. Clarke transformation is used to obtain voltage and current components in $\:\alpha\:\beta\:$ frame. These components control the active and reactive power expressed as

1 $$\:P=\frac{3}{2}\left({V}_{\alpha\:}{I}_{\alpha\:}+{V}_{\beta\:}{I}_{\beta\:}\right)$$

2 $$\:Q=\frac{3}{2}(-{V}_{\alpha\:}{I}_{\beta\:}+{V}_{\beta\:}{I}_{\alpha\:})$$

Both power components have an LPF for noise and harmonic suppression. Typically, a basic first-order filter with a cutoff frequency $\:{\omega\:}_{c}$​ is used for this purpose. The chosen cut-off frequency is recommended to be one to two decades lower than the nominal frequency. This ensures effective noise suppression while maintaining a slow transient response of the delivered power, a feature desirable in electrical power systems^24^. The power control loop generates the reference currents $\:{I}_{\alpha\:}^{*}$ and $\:{I}_{\beta\:}^{*}$as defined in Eqs. (3) and (4), using the converter output voltage and the reference values for active and reactive power^25^.

3 $$\:{I}_{\alpha\:}^{*}\:=\frac{2}{3}\frac{\left({V}_{\alpha\:}\:{P}_{ref}+{V}_{\beta\:}\:{Q}_{ref}\right)}{{V}_{\alpha\:}^{2}+{V}_{\beta\:}^{2}}$$

4 $$\:{I}_{\beta\:}^{*}=\frac{2}{3}\frac{\left({V}_{\beta\:}\:{P}_{ref}-{V}_{\alpha\:}\:{Q}_{ref}\right)}{{V}_{\alpha\:}^{2}+{V}_{\beta\:}^{2}}\:$$

These current components pass through an inner current control loop to provide the modulating signals $\:{m}_{\alpha\:}$ and $\:{m}_{\beta\:}$for the converter switches. A PR controller is utilized in the inner current control loop to minimize the error between the output current and reference current. The transfer function of the PR controller is defined as^26^

5 $$\:PR\left(S\right)\:\:={k}_{p}+\frac{2\:{k}_{i}\:\zeta\:\:{\omega\:}^{\text{*}}\:S}{{S}^{2}+2\:\zeta\:\:{\omega\:}^{\text{*}}\:S+{{\omega\:}^{\text{*}}}^{2}}$$

where $\:{k}_{p}$ is the proportional gain, $\:{k}_{i}$ is the integral gain, $\:{\omega\:}^{\text{*}}$ is the angular frequency reference and $\:\zeta\:$ is the damping coefficient.

## Optimization of grid-feeding control

**Fig. 4:**
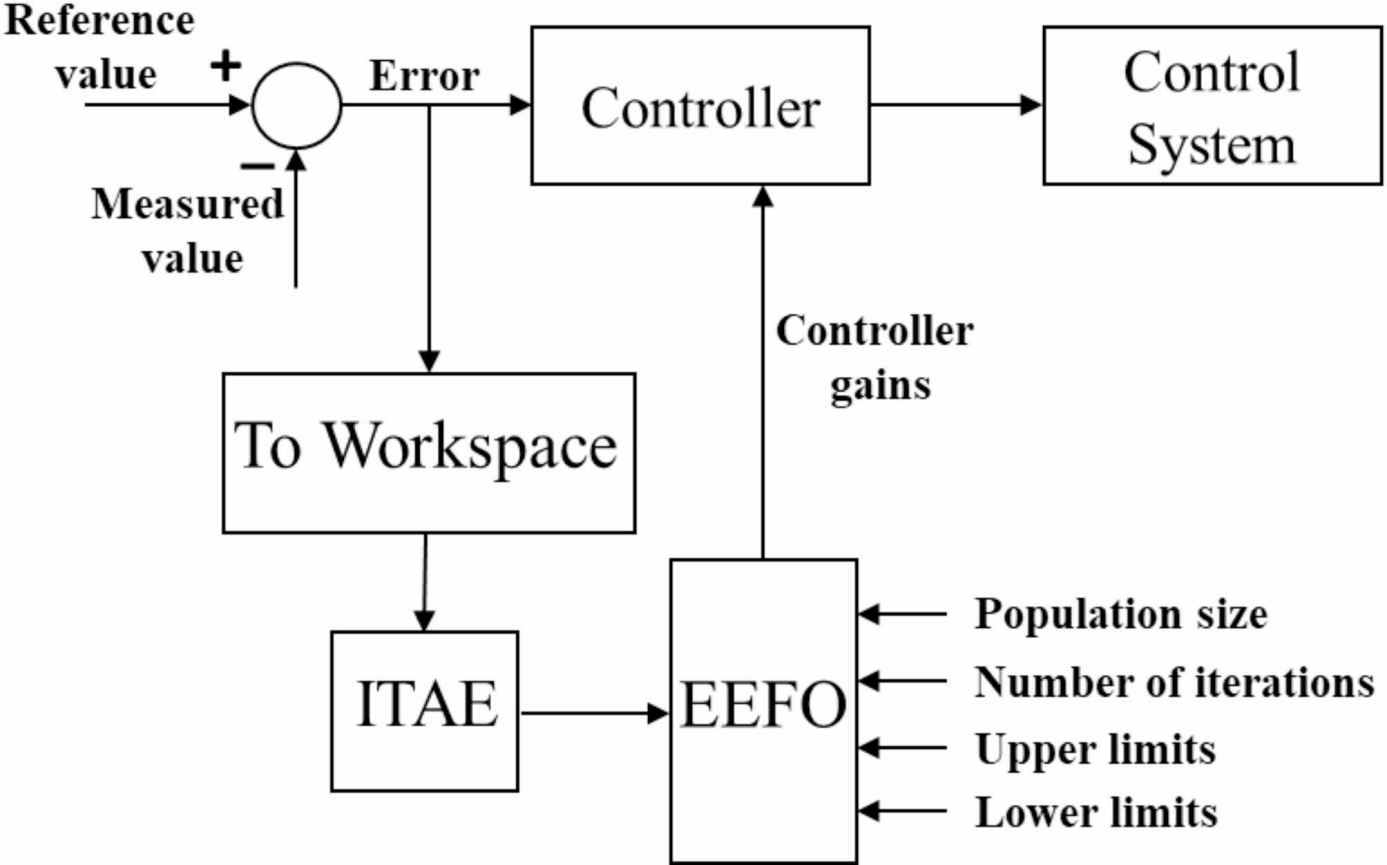
Optimization process working scheme.

The proposed optimization algorithm is employed to tune the controllers of the grid-feeding unit. Four FF criteria are commonly used in literature which are Integral Square Error (ISE), Integral Absolute Error (IAE), Integral Time Square Error (ITSE), and Integral Time Absolute Error (ITAE). However, ITAE is the most extensively applied FF criterion compared to its competitors because of its ease of implementation, realistic error indexing, and superior results^21,22^. The ISE and ITSE are employed to square the error, resulting in substantial perturbations in results even for very minor changes in the error signal, and so producing unrealistic results. Furthermore, as the absolute error is continuously multiplied by time, the ITAE generates more realistic error indexing than the IAE. Given the major properties of the ITAE criterion, it is chosen as the FF to be minimized in this work. The optimization problem can be described as follows

6 $$\:let\:k=\left[{k}_{pP}\:{k}_{iP}\:{k}_{pQ}\:{k}_{iQ}\right]Minimize\:FF=\underset{0}{\overset{{\infty\:}}{\int\:}}t.\left|{e}_{\text{P}}\right|dt+\underset{0}{\overset{{\infty\:}}{\int\:}}t.\left|{e}_{\text{Q}}\right|dtVariable\:limits=\left\{\begin{array}{c}{k}_{pP}=0\\\:0.35\le\:{k}_{iP}\le\:0.65\\\:4.2\le\:{k}_{pQ}\le\:7.8\\\:10.5\le\:{k}_{iQ}\le\:19.5\end{array}\right.$$

where $\:{e}_{\text{P}}$ and $\:{e}_{\text{Q}}$ are the errors in the active and reactive power components, respectively. Figure 4 shows the optimization process working scheme.

## Control of grid-forming converter

The grid-forming converter adopts a hierarchical control framework with primary and secondary control levels. The primary control level consists of voltage and current control loops, along with a droop control loop. It is responsible for power sharing and ensuring stable operation in islanded mode. The secondary control includes voltage amplitude and frequency restoration loops for maintaining the operating voltage and frequency at nominal values. It also includes a synchronization loop for a seamless transition from islanded into grid-connected mode.

### ***A. Primary control level***

#### 1) Droop control with virtual impedance

The primary objectives of droop controller are to ensure the stable operation of the microgrid, facilitate active and reactive power sharing among parallel DGs, and support plug-and-play capability^27–29^. Droop control does not require any external communication links between converters, which is a significant benefit. Furthermore, its simple implementation,, relying only on local voltage and current measurements, facilitates seamless plug-and-play operation. As a result, it improves system redundancy and facilitates future expansions. The proposed droop controller is described by the following equations .

7 $$\:{\upomega\:}={{\upomega\:}}^{\text{*}}-{m}_{p}\:P-{m}_{PP}\frac{dP}{dt}$$

8 $$\:V={V}^{\text{*}}-{n}_{q}\:Q$$

where $\:\omega\:$ and $\:V$ represent the converter’s output angular frequency and voltage amplitude, respectively. $\:{\omega\:}^{\text{*}}$and $\:{V}^{\text{*}}$ are the nominal values for angular frequency and voltage amplitude, respectively. $\:P$ and $\:Q$ are the calculated active and reactive power, as determined by (1) and (2). $\:{m}_{p}$ and $\:{n}_{q}\:$ denote proportional droop parameters for the frequency and voltage, respectively, and $\:{m}_{PP}$ is a derivative parameter added to improve the transient response.

A virtual impedance control loop is used to control the behavior of the output impedance of the converter, which improves power-sharing accuracy^23,24^. The virtual impedance is incorporated into the voltage reference signal as an additional variable, derived from the output current, as shown below.

9 $$\:{V}_{ref}=Vsin\left(\phi\:\right)-({R}_{V}{i}_{o}+{L}_{V}\frac{d{i}_{o}}{dt})$$

Here $\:\phi\:$ represents the integral of Eq. (7) over time, $\:{i}_{o}$ denotes the converter’s output current. $\:{R}_{V}$ and $\:{L}_{V}$ correspond to the resistive and inductive parts of the virtual impedance, respectively.

10 $$\:{Z}_{V}={R}_{V}+j\:{L}_{V}$$

The virtual impedance $\:{Z}_{V}$ is designed to have inductive behavior in order to control active and reactive power using the equations of droop controller presented in (6) and (7). As the *α* component leads the *β*component by 90°, the time derivative term can be calculated using cross-coupling as follows in (11) and (12)^30,31^.

11 $$\:{Z}_{V}{i}_{o\alpha\:}={R}_{V}{i}_{o\alpha\:}+{\omega\:}^{\text{*}}{L}_{V}{i}_{o\beta\:}$$

12 $$\:{Z}_{V}{i}_{o\beta\:}={R}_{V}{i}_{o\beta\:}-{\omega\:}^{\text{*}}{L}_{V}{i}_{o\alpha\:}$$

**Fig. 5:**
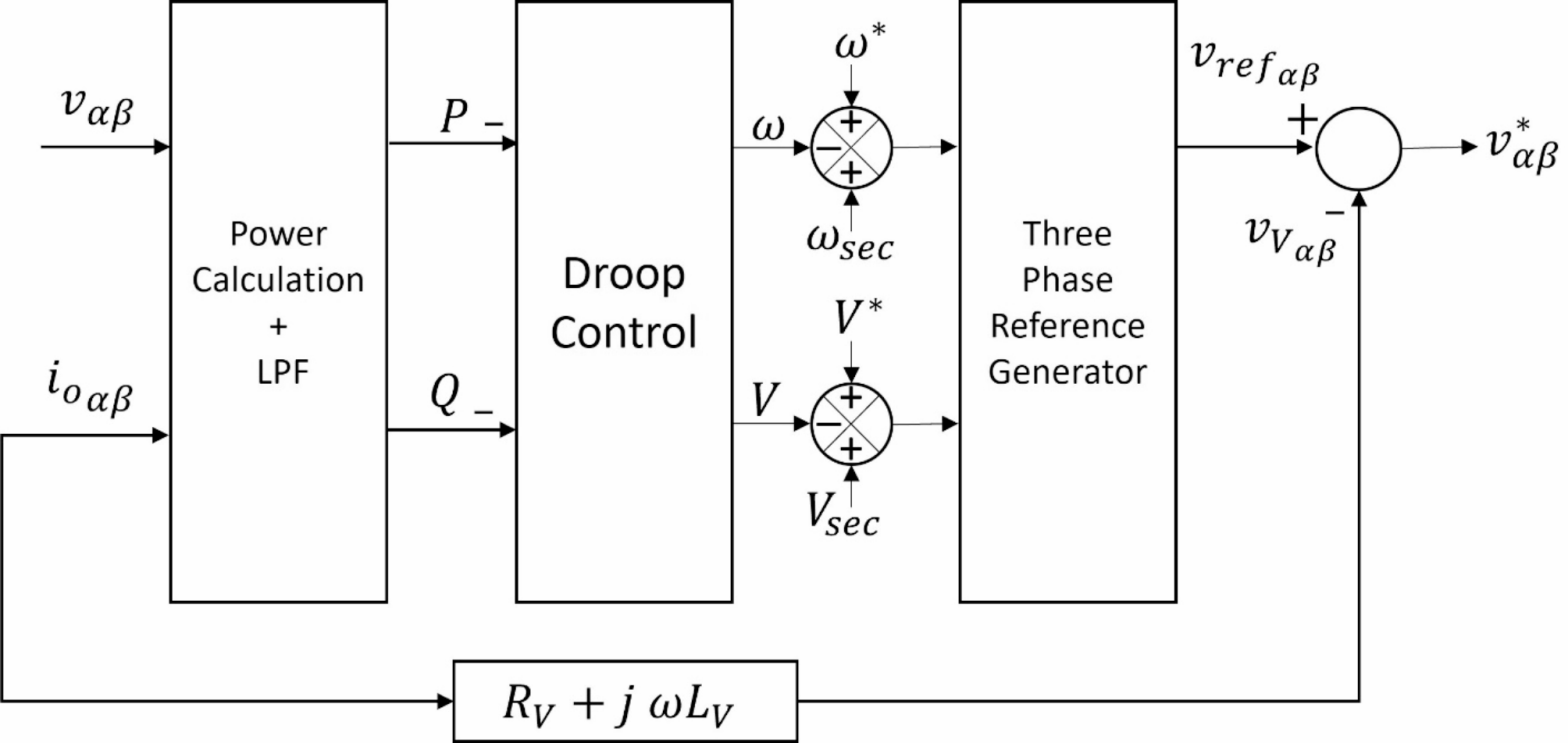
block diagram of virtual impedance-based droop control.

Figure 5 depicts the droop controller with the virtual impedance loop.

#### *2) Inner control loops*

**Fig. 6:**
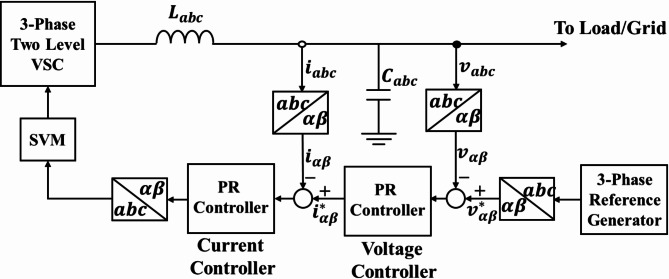
Inner control loops block diagram for 3-phase VSC.

Figure 6 illustrates the inner control loops of a three-phase space vector modulation (SVM) converter with an LC filter. PR compensators tuned to the fundamental frequency are utilized in current and voltage control loops.

The three-phase reference generator provides the voltage references $\:{v}_{\alpha\:}^{*}$ and $\:{v}_{\beta\:}^{*}$ based on the measured line currents and output voltage of the converter in $\:\alpha\:\beta\:$ coordinates, as shown in Fig. 6. These reference voltage signals are used by the voltage control loop to produce reference current signals, which are then used by the current control loop to generate modulating signals for the converter.

### ***B. Secondary control level***

**Fig. 7:**
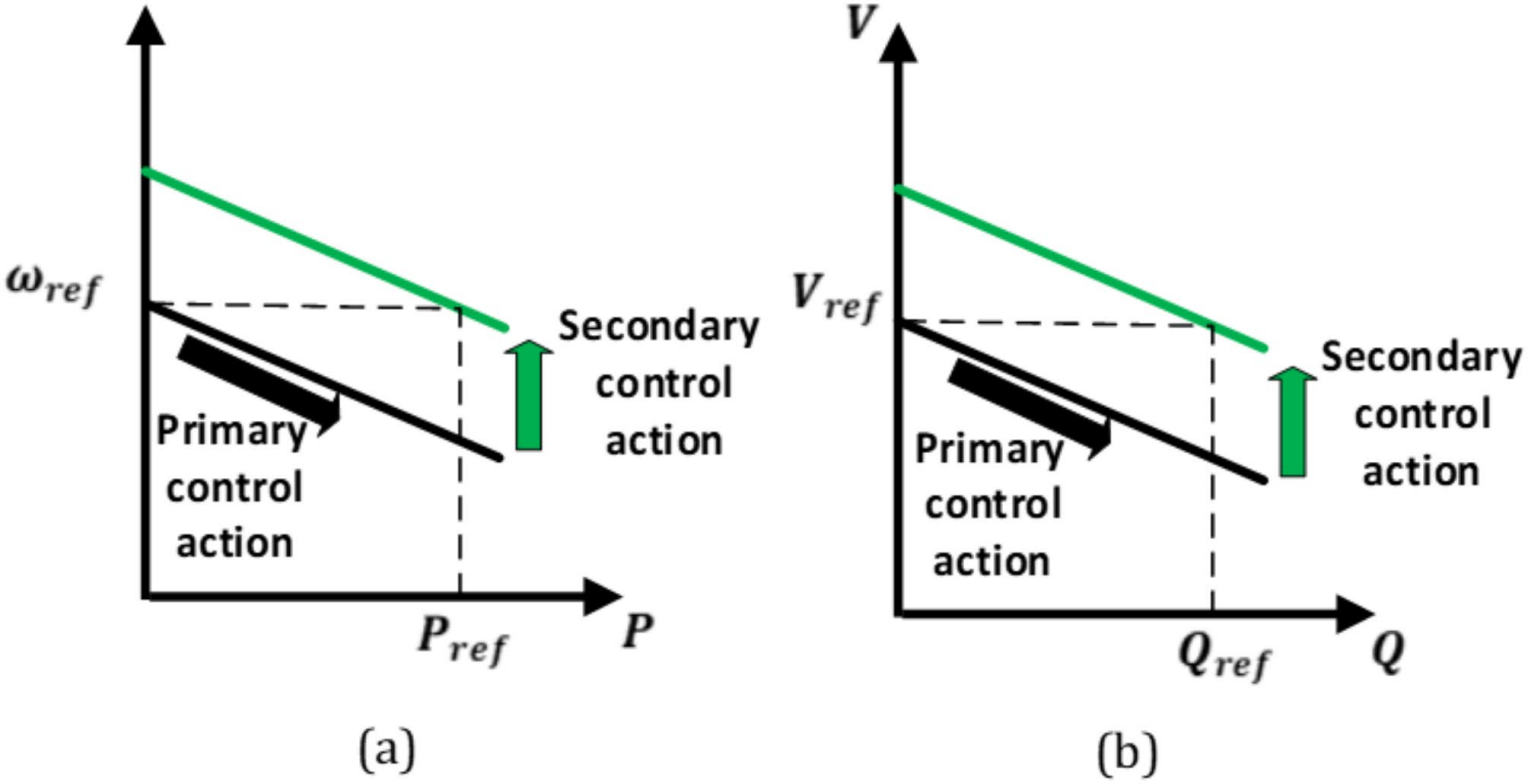
Effects of primary and secondary controllers.

The secondary controller restores the nominal voltage amplitude and frequency by adding a compensating term that adjusts the droop functions to their original values, while maintaining the power-sharing achieved by the primary control. It also includes a synchronization loop to prepare the microgrid for a seamless connection with the utility grid. The primary and secondary control actions are depicted in Fig. 7.

#### *1) Voltage amplitude and frequency restoration*

The secondary control compares the nominal PCC voltage amplitude and frequency values with the measured ones. The generated error is processed using a PI controller. As a result, the deviations in voltage amplitude and frequency are eliminated. The compensating terms for voltage amplitude and frequency are expressed as follows.

13 $$\:{V}_{sec}={k}_{pvs}\left({V}^{*}-{V}_{PCC}\right)+{k}_{ivs}\int\:({V}^{*}-{V}_{PCC})\:dt$$

14 $$\:\:{\omega\:}_{sec}={k}_{p\omega\:}\left({\omega\:}^{\text{*}}-{\omega\:}_{PCC}\right)+{k}_{i\omega\:}\int\:\left({\omega\:}^{\text{*}}-{\omega\:}_{PCC}\right)\:dt$$

where $\:{k}_{pV}$, $\:{k}_{iV}$ and$\:\:{k}_{p\omega\:}$, $\:{k}_{i\omega\:}$ are the parameters of the PI controllers for the voltage amplitude and frequency restoration loops, respectively.

#### *2) Synchronization loop*

The secondary control additionally incorporates a synchronization loop to prepare the microgrid for seamless grid connection. An STS connects the PCC in the microgrid to the utility grid. The utility grid and PCC voltages are $\:{V}_{g}$ and $\:{V}_{mg}$, respectively. Without an appropriate synchronization method, the mismatches in voltage between $\:{V}_{mg}$ and $\:{V}_{g}$ would cause substantial inrush currents, which would be hazardous to microgrid operations. The grid synchronization approach is vital for reducing excessive inrush currents and ensuring continuous operation of critical loads. Synchronization between $\:{V}_{mg}$ and $\:{V}_{g}$ in voltage amplitude, phase, and frequency, is essential to ensure a smooth and successful reconnection. Voltage mismatches between the utility grid and PCC are handled by the synchronization controller to produce synchronization correction signals $\:\varDelta\:{\omega\:}_{s}$ and $\:\varDelta\:{V}_{s}$. The synchronization loop aims to minimize voltage differences between the utility grid and PCC^25–27^. In a three-phase microgrid, the voltage phase angle error is estimated by taking the cross product of the voltage vectors between utility grid and PCC as follows^32^.

15 $$\:{e}_{\theta\:}={V}_{mg}\:{V}_{g}\text{sin}\left({\theta\:}_{g}-{\theta\:}_{mg}\right)\:=-{V}_{{g}_{\alpha\:}}\:{V}_{m{g}_{\beta\:}}+{V}_{{g}_{\beta\:}}\:{V}_{m{g}_{\alpha\:}}$$

According to (15), $\:{e}_{\theta\:}$ is always identically equal to zero if both the angle difference and the angular frequency difference are zero. The voltage amplitude error is described as

16 $$\:{e}_{V}={V}_{g}-{V}_{mg}=\sqrt{{V}_{{g}_{\alpha\:}}^{2}+{V}_{{g}_{\beta\:}}^{2}}-\sqrt{{V}_{m{g}_{\alpha\:}}^{2}+{V}_{m{g}_{\beta\:}}^{2}}\:$$

**Fig. 8:**
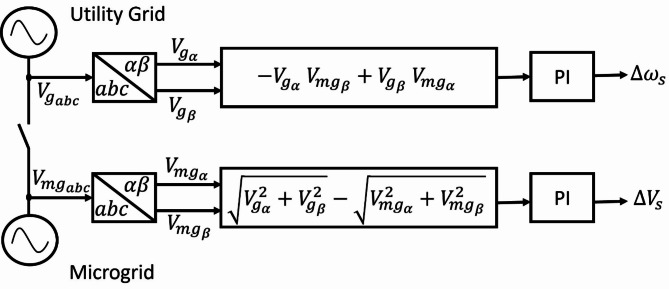
Synchronization control loop.

The errors $\:{e}_{\theta\:}$ and $\:{e}_{V}$ are processed by PI controllers which generate the correction signals required for synchronization. The $\:\varDelta\:{\omega\:}_{s}$ and $\:\varDelta\:{V}_{s}$synchronization correction signals are given as^32^

17 $$\:\varDelta\:{\omega\:}_{s}=\left({k}_{ps}+\frac{{k}_{is}}{s}\right)\left(\frac{-{V}_{{g}_{\alpha\:}}\:{V}_{m{g}_{\beta\:}}\:+{V}_{{g}_{\beta\:}}\:{V}_{m{g}_{\alpha\:}}}{{V}_{mg}\:{V}_{g}}\right)$$

18 $$\:\varDelta\:{V}_{s}=\left({k}_{pvs}+\frac{{k}_{ivs}}{s}\right)\left(\sqrt{{V}_{{g}_{\alpha\:}}^{2}+{V}_{{g}_{\beta\:}}^{2}}-\sqrt{{V}_{m{g}_{\alpha\:}}^{2}+{V}_{m{g}_{\beta\:}}^{2}}\right)$$

where $\:{k}_{ps}$, $\:{k}_{pvs}$ denote the proportional parameters while $\:{k}_{is}$, $\:{k}_{ivs}$ denote the integral parameters. Figure 8 shows a block diagram for the synchronization control loop.

## Optimization of grid-forming control

### ***A. Primary control optimization***

The proposed EEFO algorithm is applied for optimization of primary-level controllers. A multi-objective function is utilized to improve steady-state and dynamic performance for different operating scenarios. The multi-objective function minimizes the arithmetic sum of the following: ITAE of $\:\alpha\:$ and $\:\beta\:$ voltage components, ITAE of $\:\alpha\:$ and $\:\beta\:$ current components, ITAE of voltage amplitude, and ITAE of frequency. The optimization problem can be formulated as follows.

19 $$\begin{aligned}\:consider\:k=\left[{m}_{p}\:{m}_{pp}\:{n}_{q}\:{k}_{pv}\:{k}_{iv}\:{\zeta\:}_{v}\:{k}_{pi}\:{k}_{ii}\:{\zeta\:}_{i}\right]\: Minimize\:FF \cr \:={w}_{1}\underset{0}{\overset{{\infty\:}}{\int\:}}t.\left|{e}_{{v}_{\alpha\:}}\right|dt+{w}_{2}\underset{0}{\overset{{\infty\:}}{\int\:}}t.\left|{e}_{{v}_{\beta\:}}\right|dt+{w}_{3}\underset{0}{\overset{{\infty\:}}{\int\:}}t.\left|{e}_{{i}_{\alpha\:}}\right|dt \cr \:\:\:\:+{w}_{4}\underset{0}{\overset{\infty\:}{\int\:}}t.\left|{e}_{{i}_{\beta\:}}\right|dt+{w}_{5}\underset{0}{\overset{\infty\:}{\int\:}}t.\left|{e}_{V}\right|dt+{w}_{6}\underset{0}{\overset{\infty\:}{\int\:}}t.\left|{e}_{f}\right|dt \cr \:Variable\:limits=\left\{\begin{array}{c}\begin{array}{c}100\times\:{10}^{-6}\le\:{m}_{p}\le\:150\times\:{10}^{-6}\\\:7\times\:{10}^{-6}\le\:{m}_{pp}\le\:13\times\:{10}^{-6}\\\:7\times\:{10}^{-4}\le\:{n}_{q}\le\:1.4\times\:{10}^{-3}\\\:0.06\le\:{k}_{pv}\le\:0.14\\\:0.06\le\:{k}_{iv}\le\:0.14\\\:0.006\le\:{\zeta\:}_{v}\le\:0.014\end{array}\\\:9.5\le\:{k}_{pi}\le\:14.5\\\:150\le\:{k}_{ii}\le\:250\\\:0.07\le\:{\zeta\:}_{i}\le\:0.13\end{array}\right.\end{aligned}$$

where $\:{w}_{i}$ represents a weight coefficient determining the priority of each term in the multi-objective function; $\:{e}_{{v}_{\alpha\:}}$ and $\:{e}_{{v}_{\beta\:}}$ denote the error in $\:\alpha\:$ and $\:\beta\:$ voltage component; and $\:{e}_{{i}_{\alpha\:}}$ and $\:{e}_{{i}_{\beta\:}}$ denote the error in $\:\alpha\:$ and $\:\beta\:$ current component; $\:{e}_{V}$ and $\:{e}_{f}$ denote the error in voltage amplitude and frequency, respectively.

### ***B. Secondary control optimization***

#### *1) Restoration loops optimization*

EEFO optimization algorithm is used to design the secondary control PI compensators. The optimization goal is to restore the nominal microgrid voltage and frequency with minimum settling time and overshoot. This multi-objective function can be given as^3^.

20 $$\begin{aligned}\:let\:k=\left[{k}_{pvr}\:{k}_{ivr}\:{k}_{p\omega\:r}\:{k}_{i\omega\:r}\:\right] \cr \:Minimize\:FF={w}_{1}\:{t}_{{s}_{V}}+{w}_{2}\:{t}_{{s}_{f}}+{w}_{3}\:{os}_{V}+{w}_{4}\:o{s}_{f} \cr \:Variable\:limits=\left\{\begin{array}{c}0.07\le\:{k}_{pvr}\le\:0.14\\\:18\le\:{k}_{ivr}\le\:125\\\:{k}_{p\omega\:r}=0\\\:6\le\:{k}_{i\omega\:r}\le\:19\end{array}\right.\end{aligned}$$

where $\:{t}_{{s}_{V}}$ and $\:{t}_{{s}_{f}}$ denote the settling times of the microgrid’s voltage amplitude and frequency, respectively, and $\:{os}_{V}$ and $\:{os}_{f}$ represent their maximum overshoot.

2) *Synchronization loop optimization*.

IEEE Standard (1547–2018) recommends the requirements for the reconnection of DGs with the utility grid^33–35^.

^35^. However, to ensure seamless reconnection without large inrush currents, the synchronization process should be subjected to more rigid constraints^36,37^. To achieve this goal, the synchronization controllers are optimized by the proposed algorithm. The objective function minimizes the ITAE in voltage amplitude and phase angle subject to three constraints. The optimization problem can be described as.

21 $$\begin{aligned}\:let\:k=\left[{{k}_{pvs}\:{k}_{ivs}\:k}_{p\omega\:s}\:{k}_{i\omega\:s}\:\:\right] \cr \:Minimize\:FF=\underset{0}{\overset{{\infty\:}}{\int\:}}t.\left|{e}_{{V}_{d}}\right|dt+\underset{0}{\overset{{\infty\:}}{\int\:}}t.\left|{e}_{{\theta\:}_{d}}\right|dt \cr \:Variable\:limits=\left\{\begin{array}{c}0.7\le\:{k}_{pvs}\le\:1.3\\\:70\le\:{k}_{ivs}\le\:130\\\:0.0056\le\:{k}_{p\omega\:s}\le\:0.0104\\\:1.33\le\:{k}_{i\omega\:s}\le\:2.47\end{array}\right.\: \cr \:subject\:to\:\left\{\begin{array}{c}\varDelta\:V<2\:V\\\:\varDelta\:\theta\:<1^\circ\:\\\:\varDelta\:f<0.03\:Hz\end{array}\right.\end{aligned}$$

where $\:{e}_{{V}_{d}}$ and $\:{e}_{{\theta\:}_{d}}$ represent the difference in voltage amplitude and phase angle, respectively.

## Simulation results

The proposed microgrid was built and simulated using MATLAB/SIMULINK software to validate the performance of the optimized control system. Different system parameters are provided in the appendix.

### ***A. Comparative analysis of optimization techniques under study***

To assess the performance of the proposed EEFO technique, it was compared with two popular and well-established techniques, PSO and GWO. The considered optimization techniques were tested under the same operating conditions and system parameters to optimize the specified fitness functions. To ensure a fair comparison, all algorithms were run for 40 iterations with 20 population size. Since metaheuristic algorithms begin with a randomly distributed population within a predefined search space, the study conducted 10 simulation runs for each algorithm, selecting the best (minimum) fitness function value for comparison. The convergence curves of the algorithms for different controllers are illustrated in Fig. 10. The results show that EEFO outperformed its counterparts by exhibiting superior convergence behavior and higher-quality solutions, while the other methods struggled with issues such as getting trapped in local optima or slower convergence rates. It is worth mentioning that EEFO, while effective, may involve slightly higher computational costs compared to simpler optimization methods. However, these costs can be significantly reduced by utilizing high-performance computing resources.

**Fig. 9:**
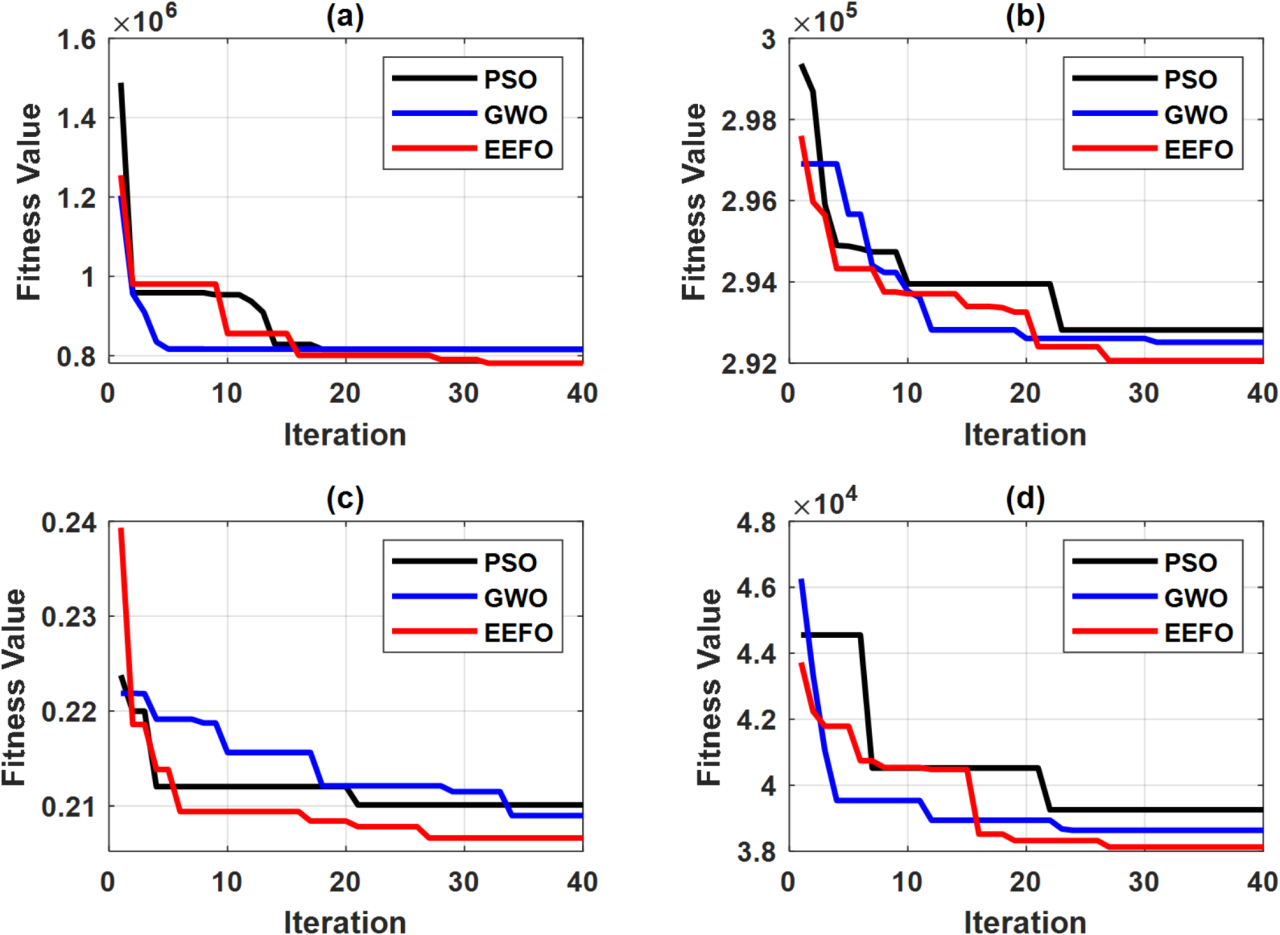
Convergence curves for optimization of (**a**) grid-feeding controllers, (**b**) primary controllers, (**c**) secondary controllers, and (**d**) synchronization controllers.

**Fig. 10:**
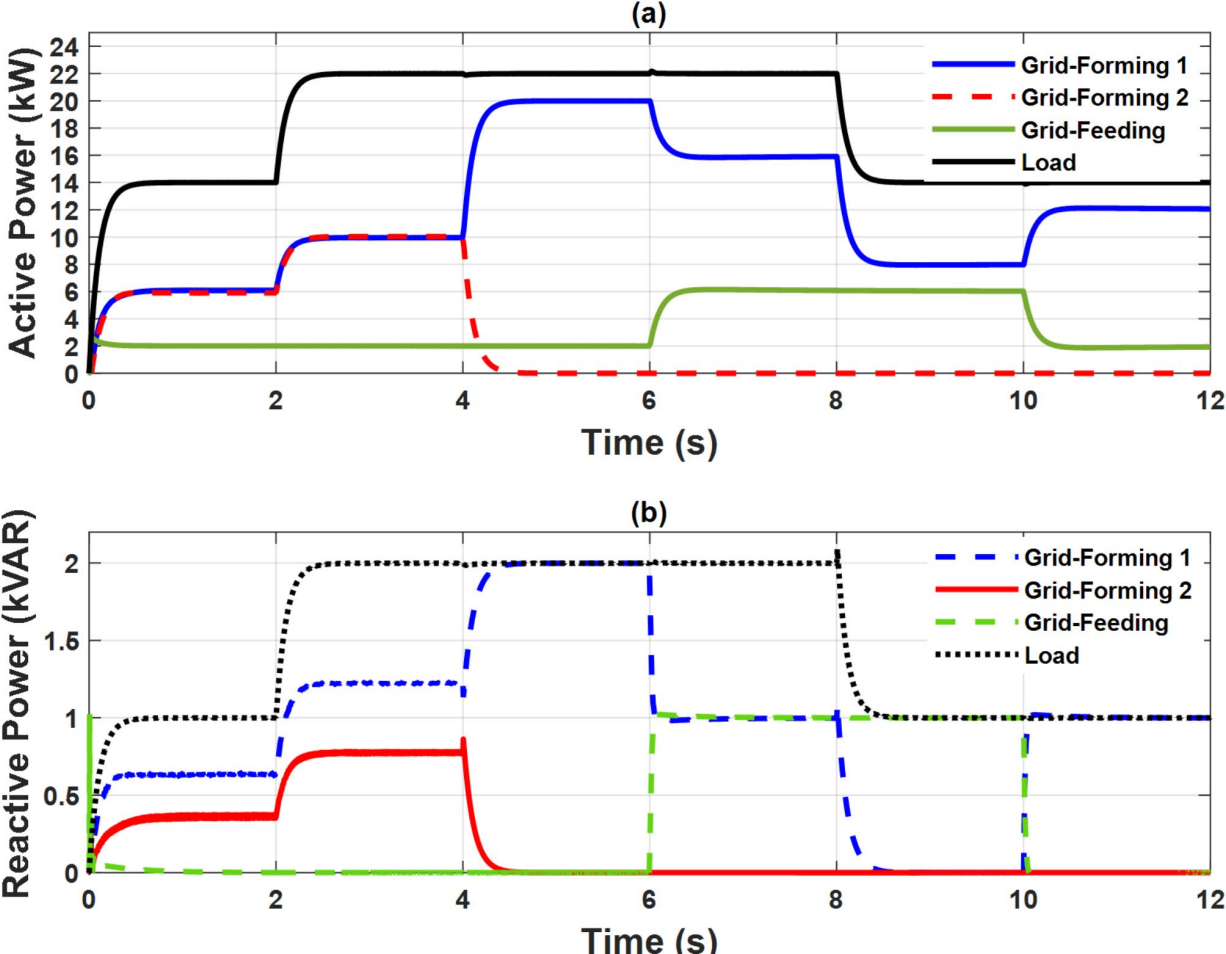
Case study 1: Power sharing (**a**) Active power and (**b**) Reactive power.

### ***B. Operational scenarios***

#### ***Case Study 1: Islanded operation***

**Fig. 11:**
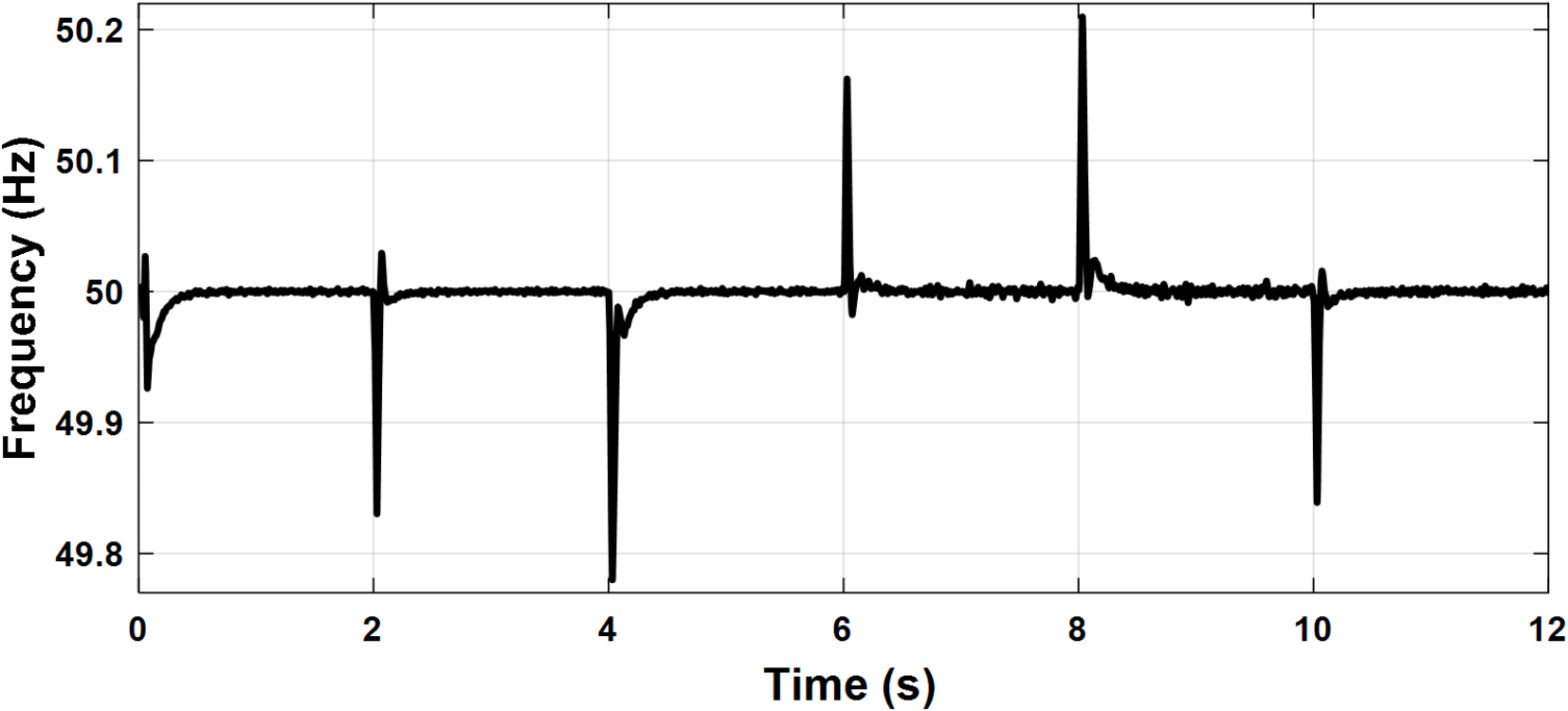
Case study 1: PCC frequency.

**Fig. 12:**
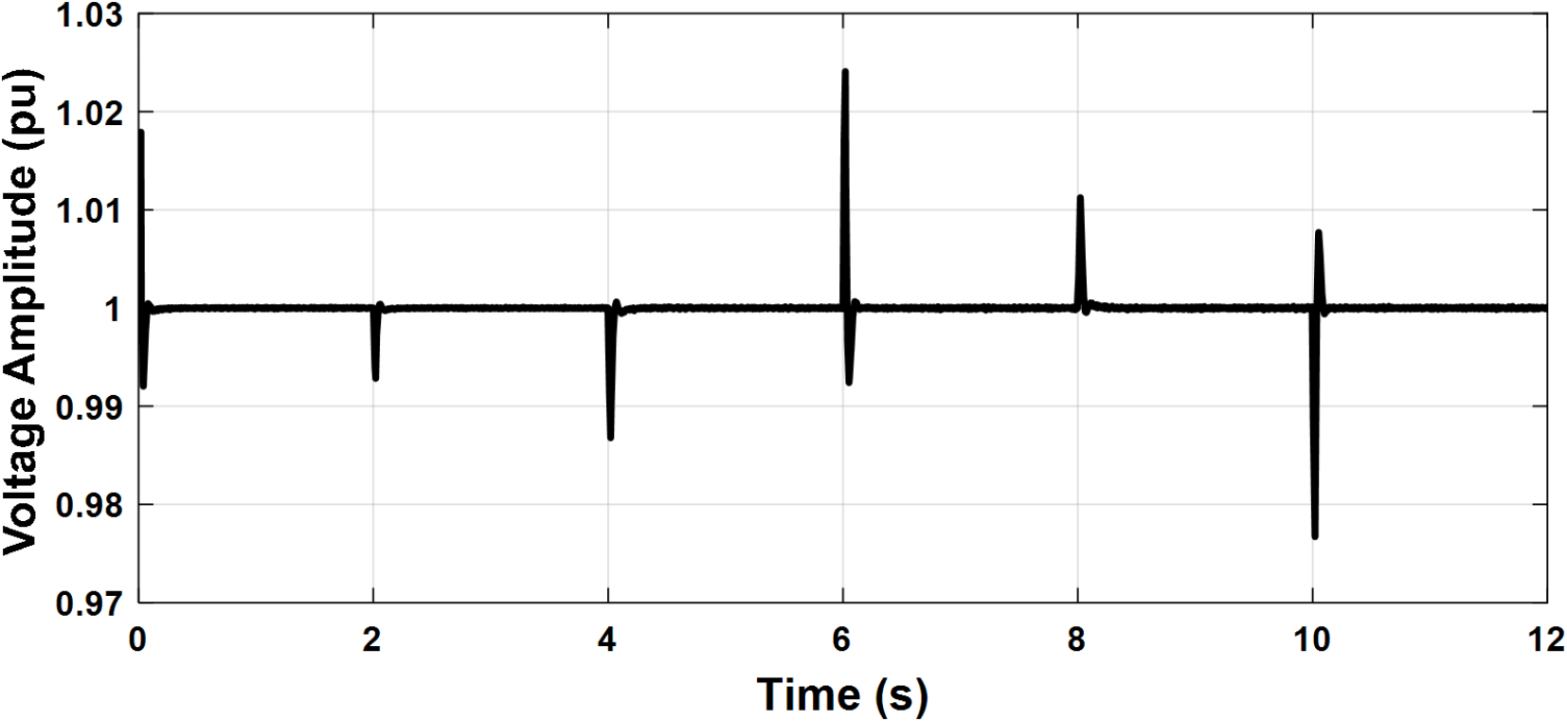
Case study 1: PCC voltage amplitude.

**Fig. 13:**
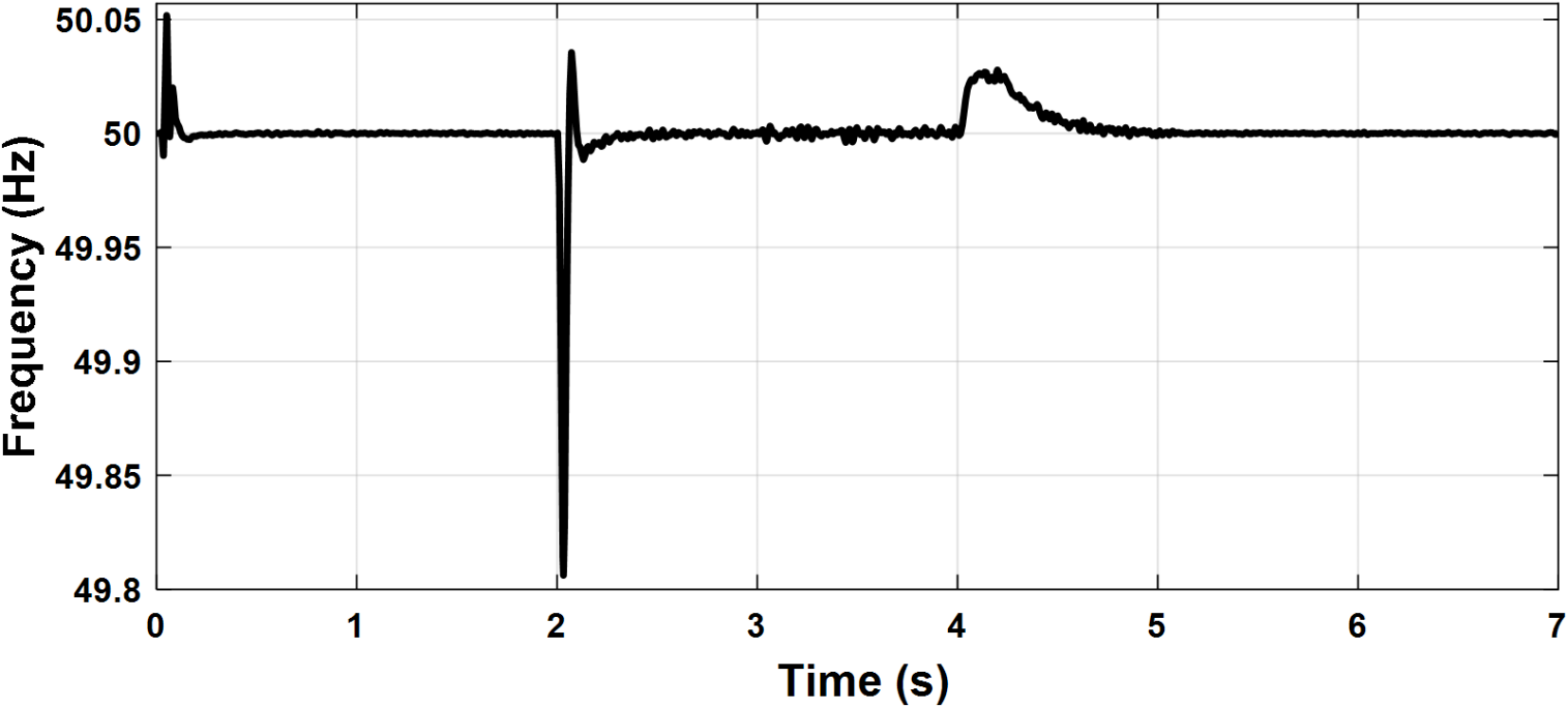
Case study 2: PCC frequency.

To illustrate the steady state and dynamic performance of the control systems, the microgrid was operated in islanded mode and subjected to a variable load as illustrated in Fig. 11. The active and reactive power sharing between the three units is also presented in the same figure. Initially, the load is (14 kW, 1 kVAR). The grid-feeding unit supplies the load with a fixed amount of 2 kW and no reactive power while the two grid-forming units share the remaining power equally. At t = 2 S, the load increased to (22 kW, 2 kVAR) and the grid forming units increased their generated power to meet the increased demand. At t = 4 S, one grid-forming unit is out of service. It can be observed that the deficit power is quickly compensated by the other grid-forming unit, ensuring continuous power supply to the load, which confirms the system’s reliability. At t = 6 S, the grid feeding unit increased its active power injection to 6 kW and participated in the reactive power supply by an amount of 1 kVAR. At t = 8 S, the load is reduced back to (14 kW, 1 kVAR). At t = 10 S, the grid-feeding unit power injection is brought back to (2 kW & 0 kVR). The grid-forming unit reacts to these changes accordingly to maintain the power balance. This test verifies the effectiveness of the droop control of the grid-forming units and the power control of the grid-feeding unit. The power-sharing between different units and load is summarized in Table 1. The PCC frequency and voltage amplitude are depicted in Figs. 12 and 13, respectively. The nominal values of frequency and voltage are maintained with little deviations at the moments of disturbance which validate the performance of the secondary control. Table 2 shows the transient response of the microgrid frequency and voltage amplitude during initiation and load change.

**Table 1:** Power sharing between DG units and load.

	Grid-feeding unit		Grid-forming unit 1		Grid-forming unit 2		Load	
Time (s)	P (kW)	Q (kVAR)	P (kW)	Q (kVAR)	P (kW)	Q (kVAR)	P (kW)	Q (kVAR)
0 → 2	2	0	6	0.64	6	0.36	14	1
2 → 4	2	0	10	1.2	10	0.8	22	2
4 → 6	2	0	20	2	0	0	22	2
6 → 8	6	1	16	1	0	0	22	2
8 → 10	6	1	8	0	0	0	14	1
10 → 12	2	0	12	1	0	0	14	1

**Table 2:** Microgrid frequency and voltage regulation.

	Frequency regulation		Voltage regulation	
	Microgrid initiation	Load change	Microgrid initiation	Load change
Max overshoot/undershoot (%)	0.15	0.42	1.8	2.41
Settling time (s)	0.5	0.53	0.2	0.25

#### ***Case study 2: Islanding event followed by reconnection to the utility grid***

**Fig. 14:**
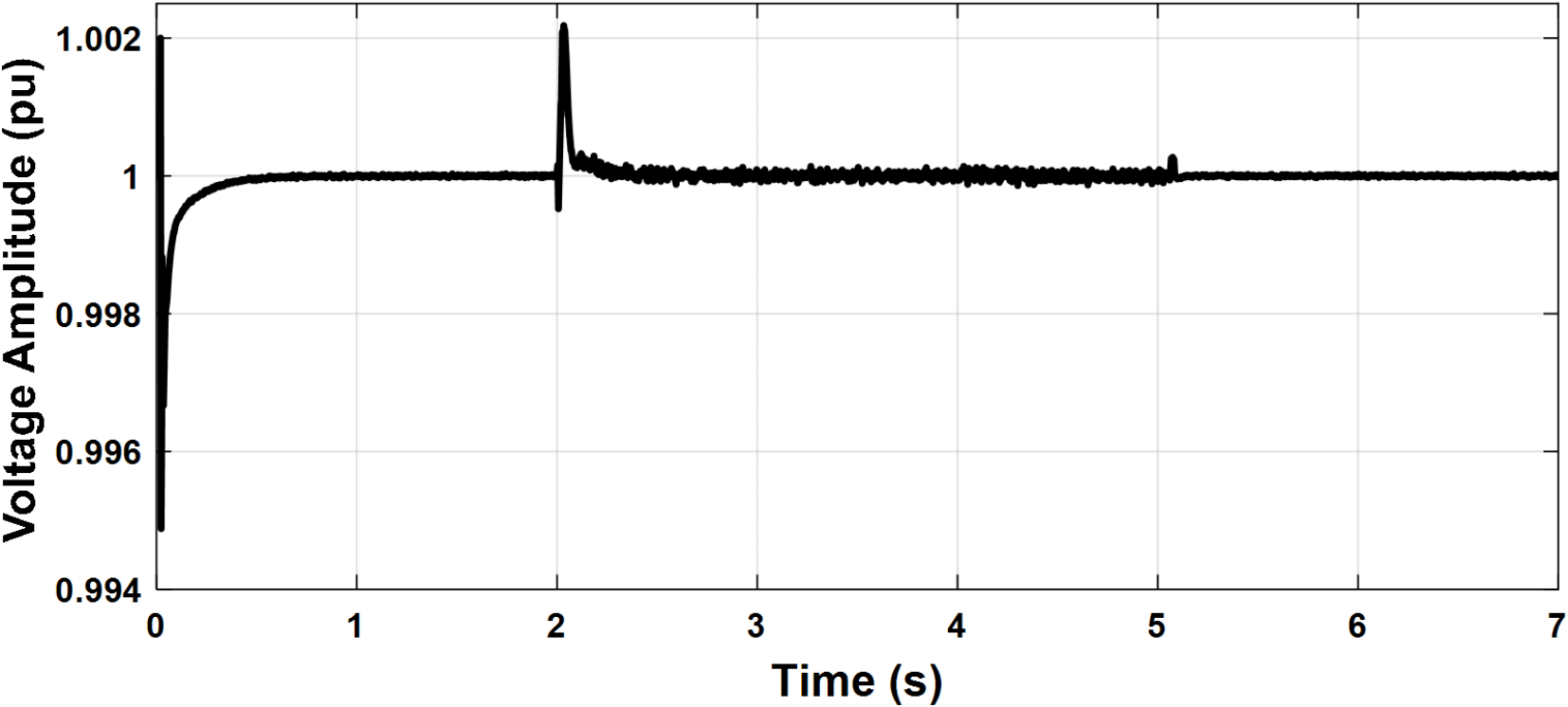
Case study 2: PCC Voltage amplitude.

**Fig. 15:**
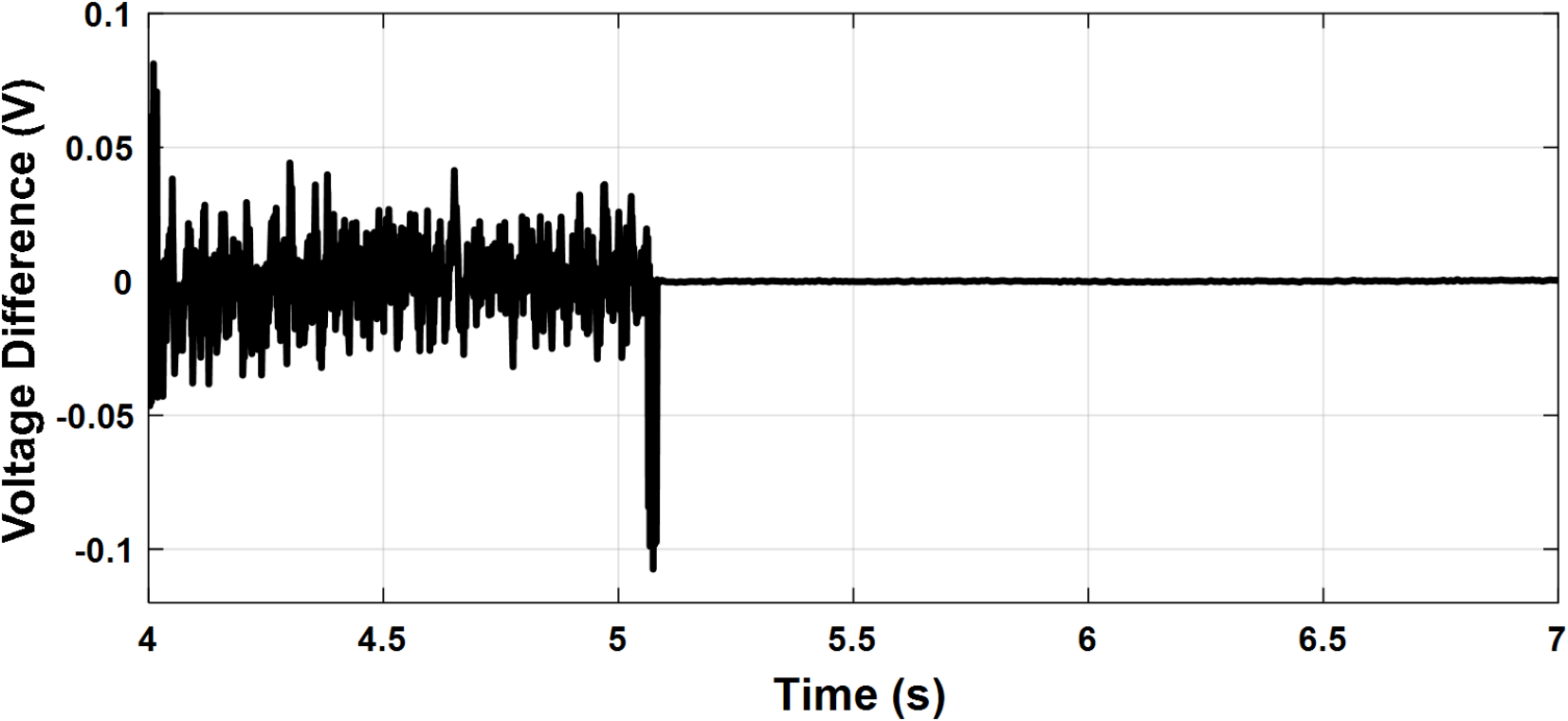
Case study 2: Voltage amplitude difference.

**Fig. 16:**
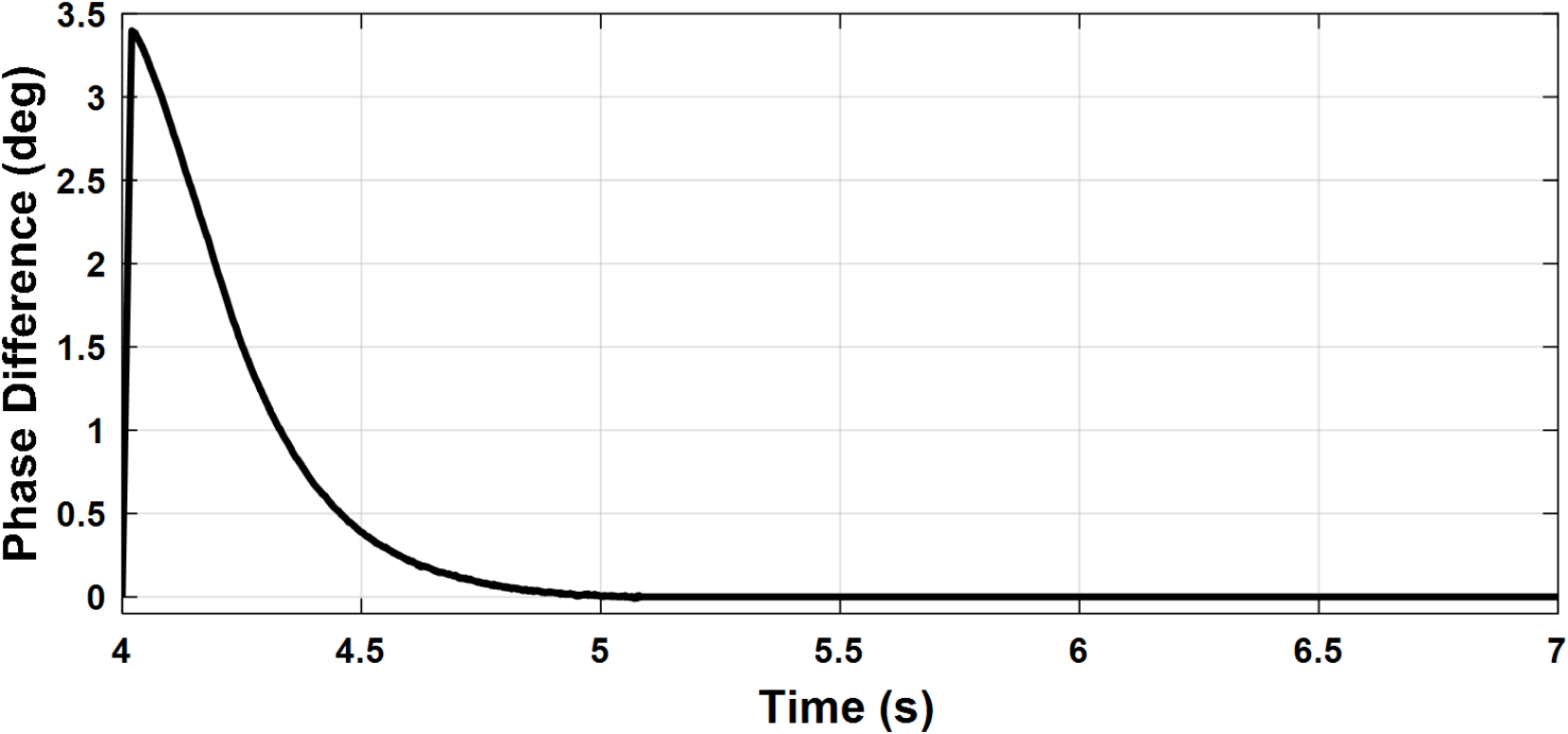
Case study 2: Phase angle difference.

**Fig. 17:**
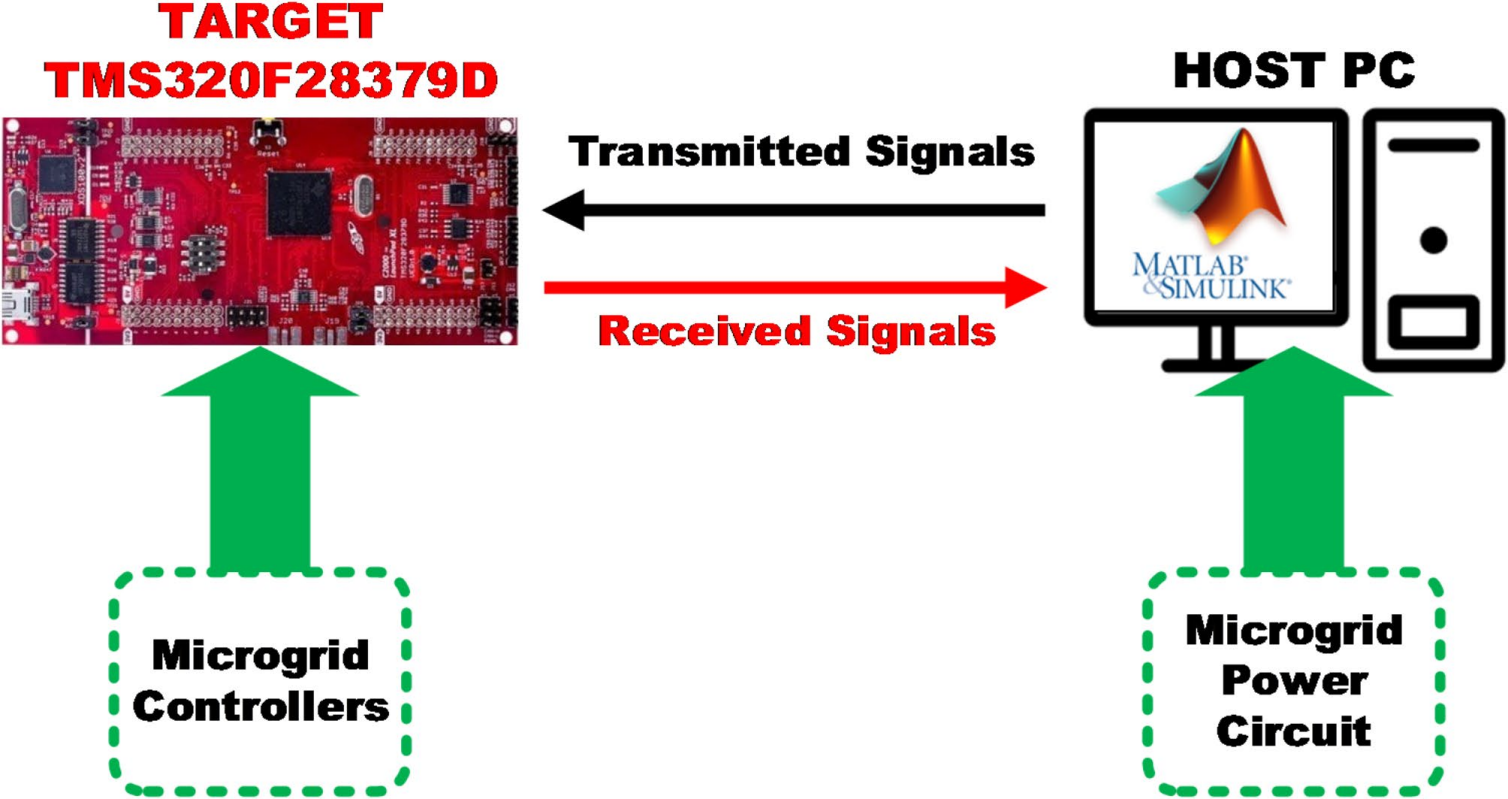
Block diagram of real-time HIL simulation setup for testing the proposed controllers.

To investigate the microgrid behavior during the transition between different modes of operation, the following scenario is simulated. The PCC frequency and voltage amplitude are shown in Figs. 14 and 15, respectively. Initially, the microgrid is grid-connected so the frequency and voltage are fixed by the stiff grid. At t = 2 s, the utility grid is disconnected and the microgrid starts to operate in islanded mode with little deviations in voltage and frequency which demonstrates a smooth transition. At t = 4 s, the synchronization loop is activated as it starts to minimize the voltage amplitude difference and phase shift between the utility grid and the microgrid as depicted in Figs. 16 and 17, respectively. Once the synchronization was achieved, the microgrid reconnected to the utility grid after approximately one second with almost no deviations in voltage and frequency which verified the seamless reconnection to the grid.

## Experimental results

The complexity of modern control systems has driven the development of efficient, cost-effective, and suitable real-time testing platforms to address critical challenges such as testing costs, failure risks, development time, safety, repeatability, availability, and system variability^38^. Hardware-in-the-loop (HIL) testing methodologies have emerged as a powerful tool, offering a real-time simulation environment to safely develop and validate complex control systems in a non-destructive and cost-efficient manner.

HIL testbeds emulate physical systems by hosting the plant model on a personal computer (PC), which interfaces with external hardware targets representing the control system. This setup allows for real-time data exchange between the Host PC and the Target hardware, facilitating rapid testing and verification under realistic load variations and operating conditions. Unlike purely numerical simulations, which often neglect disturbances, noise, and practical challenges, HIL emulation provides a more reliable and credible testing environment, ensuring the system’s performance before its deployment on actual physical plants. This approach mitigates the risk of catastrophic failures and equipment damage by isolating and validating individual components of the control system.

In this study, an HIL testbed is utilized to experimentally verify and validate the performance of EEFO-based controllers. The Host PC used for HIL emulation features an Intel(R) Core(TM) i7-5500U CPU @ 2.40 GHz and 16 GB RAM. The Target hardware, a LaunchPad XL TMS320F28379D kit, includes dual 200 MHz C28x cores, a 200 MHz real-time control co-processor (CLA), 1 MB flash memory, and 204 KB RAM. Communication between the Host PC and the Target hardware is achieved through serial communication using an XDS100v2/JTAG onboard emulator and a mini-USB cable via a virtual COM port.

The proposed HIL architecture, illustrated in Fig. 19, highlights the interaction between the Host PC and the Target hardware. On the Host PC, the microgrid’s power circuit is modeled, while the controllers are implemented on the Target TMS320F28379D kit. The connection between the Host PC and the Target DSP kit is configured using the SIMULINK support package for Texas Instruments C2000. This setup involves building the simulation model with data handling, interface, and rate transition blocks, followed by linking the Simulink model to real-world devices using Host setup blocks from the Target support toolbox.

**Fig. 18:**
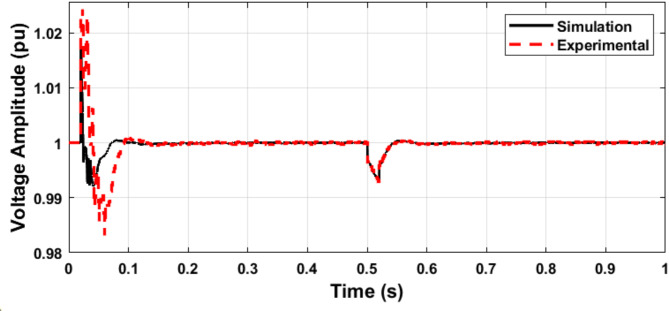
Simulation and experimental response of the microgrid voltage amplitude during load variation.

**Fig. 19:**
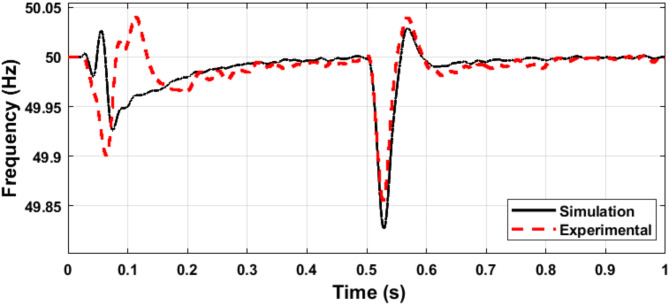
Simulation and experimental response of the microgrid frequency during load variation.

The HIL testbench comprises three primary components: the Host PC, the LAUNCHXL-F28379D Target kit from Texas Instruments with USB connectivity, and essential software packages like MATLAB/SIMULINK and Texas Instruments Code Composer Studio (used version: 10.1.0^39^). Detailed guidelines for utilizing the SIMULINK Support Package for Texas Instruments C2000 can be found in^40^.

**Fig. 20:**
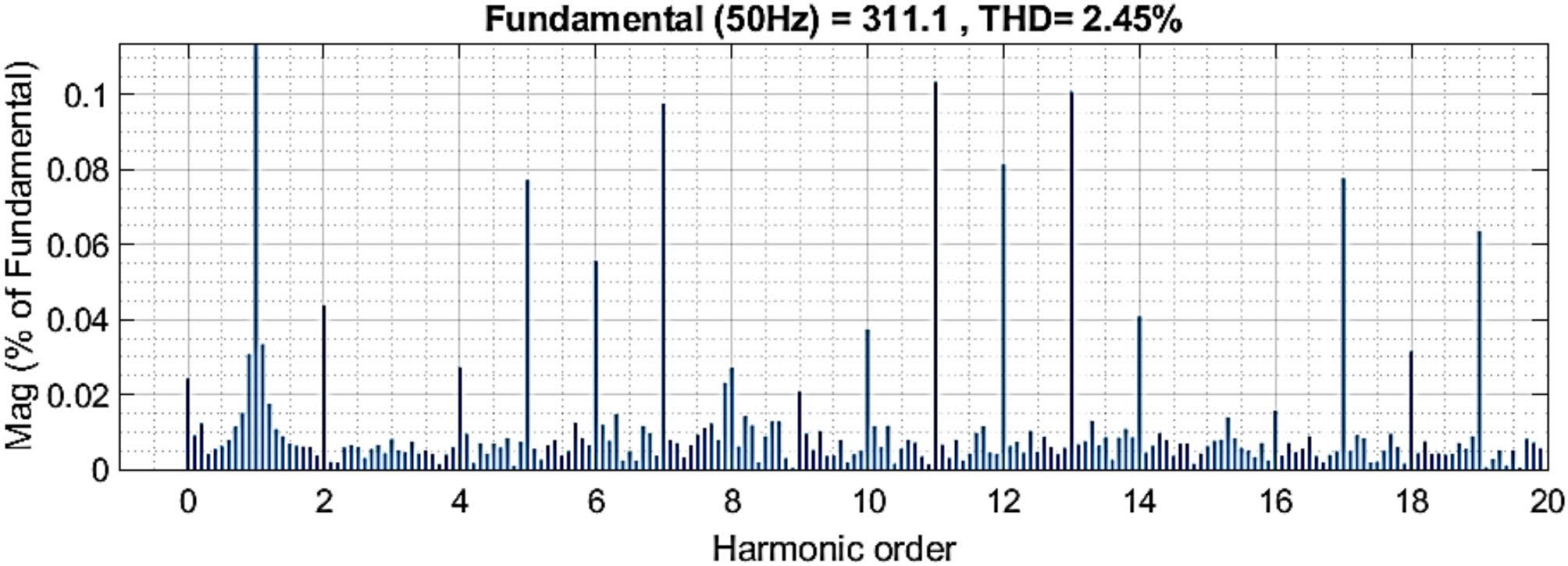
THD of microgrid voltage (simulation).

**Fig. 21:**
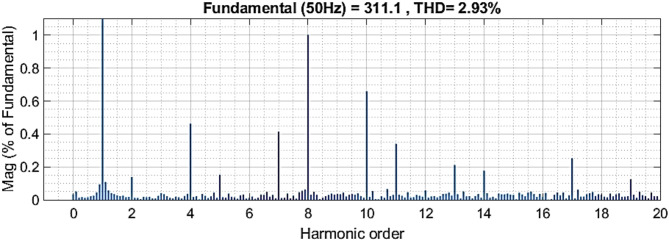
THD of microgrid voltage (Experimental).

Figures 20 and 21 show the microgrid voltage amplitude and frequency when the load is increased at t = 0.5 s from (14 kW and 1 kVAR) to (22 kW and 2 kVAR).

**Fig. 22:**
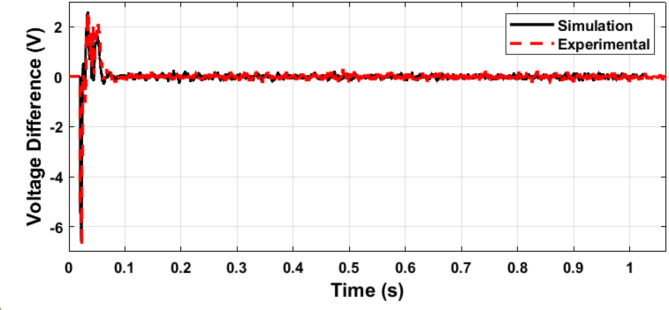
Simulation and experimental response of the voltage amplitude difference during synchronization.

**Fig. 23:**
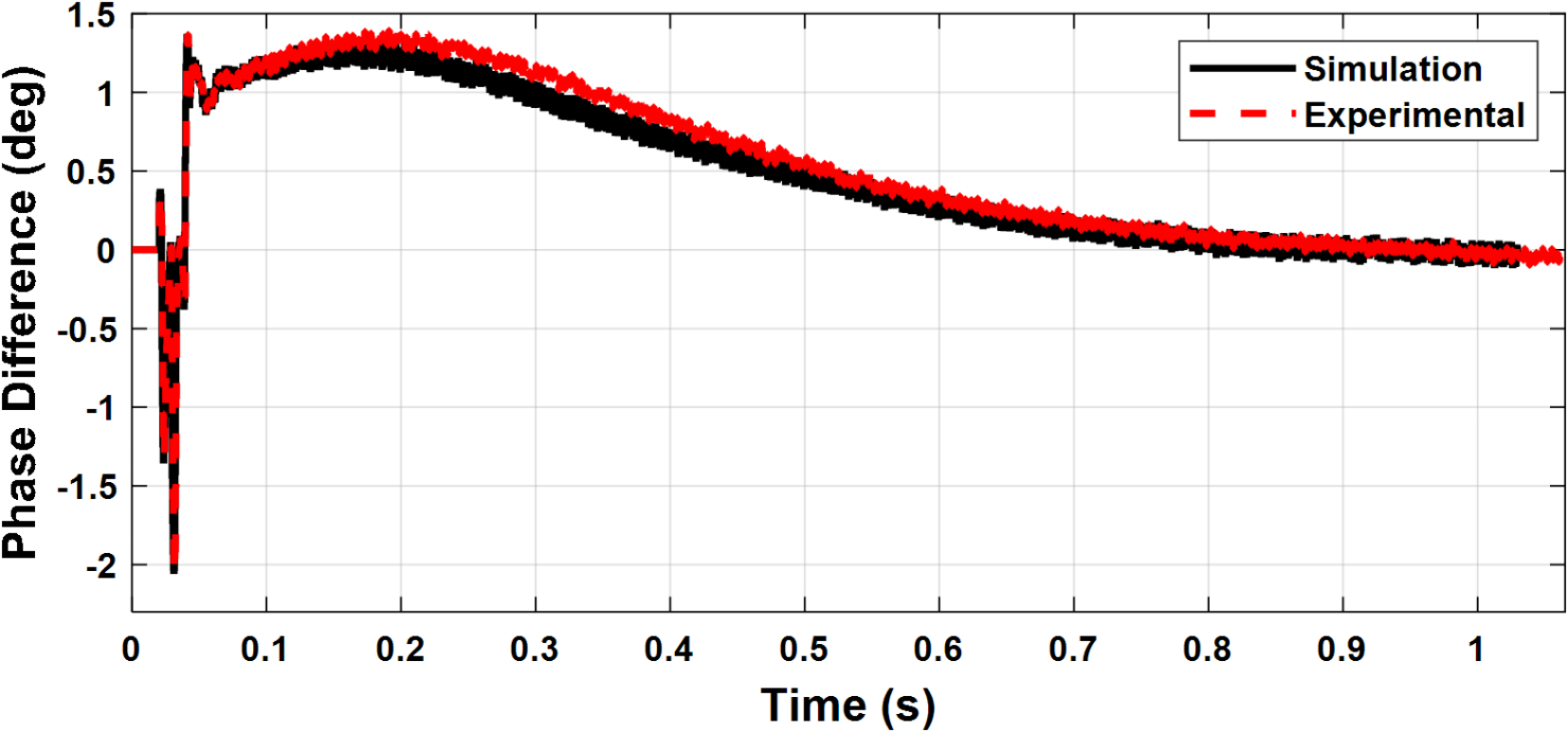
Simulation and experimental response of the phase angle difference during synchronization.

According to IEEE Standard (519–2022), the Total Harmonic Distortion (THD) at the PCC must remain below 5%^41^. As illustrated in Figs. 22 and 23, the THD achieved using the proposed controllers was 2.45% by simulation and 2.93% by experimental test. Notably, all the proposed controllers maintained THD well within the permissible limits, with a substantial safety margin, demonstrating their effectiveness and reliability.

**Fig. 24:**
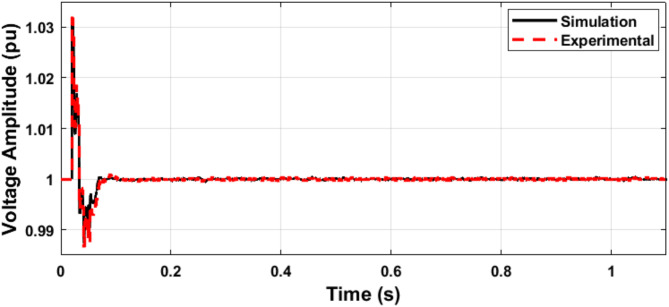
Simulation and experimental response of microgrid voltage amplitude (transition from islanded into grid-connected mode).

**Fig. 25:**
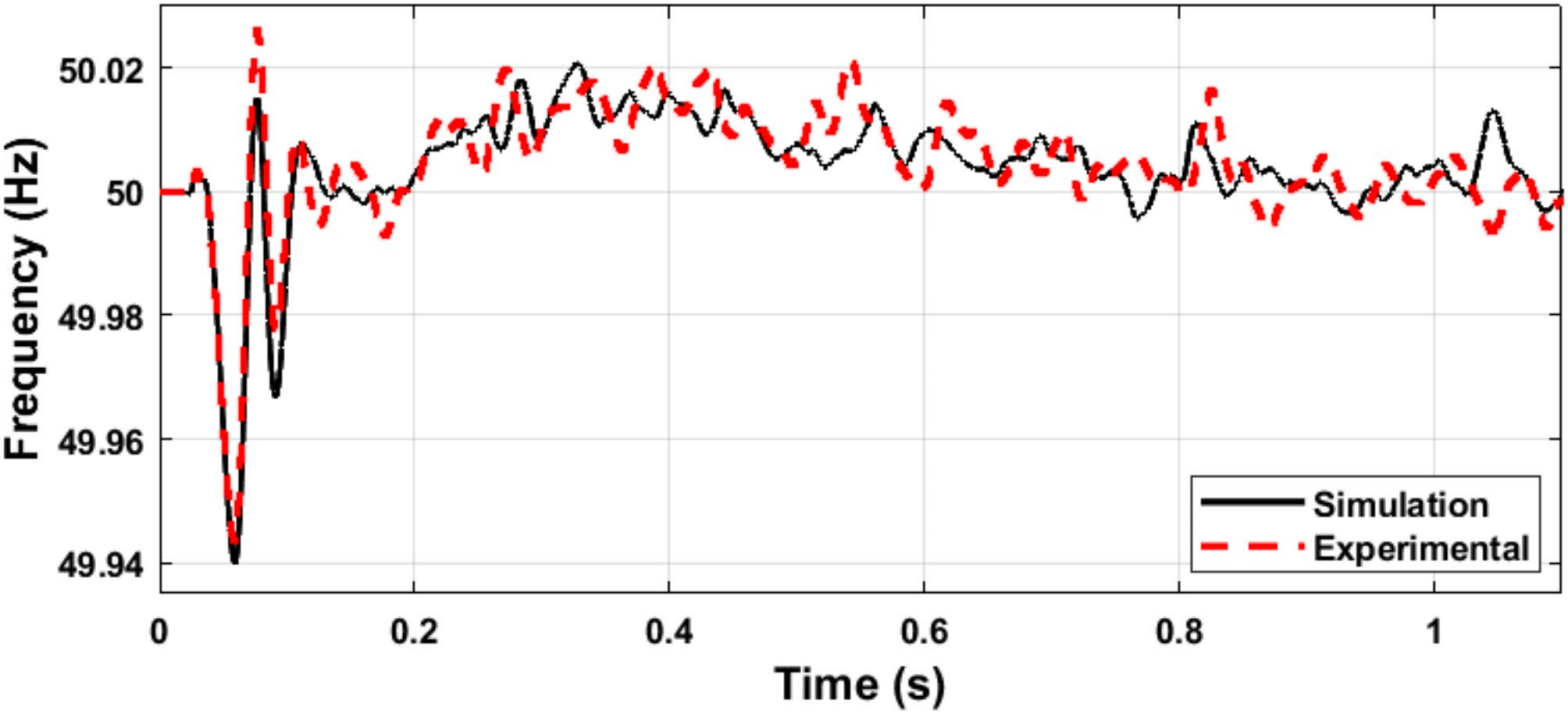
Simulation and experimental response of microgrid frequency (transition from islanded into grid-connected mode).

Figures 24 and 25 validate the synchronization controller performance in minimizing the voltage and phase differences between the microgrid and the utility grid for a seamless transition from islanded into grid-connected mode.

**Fig. 26:**
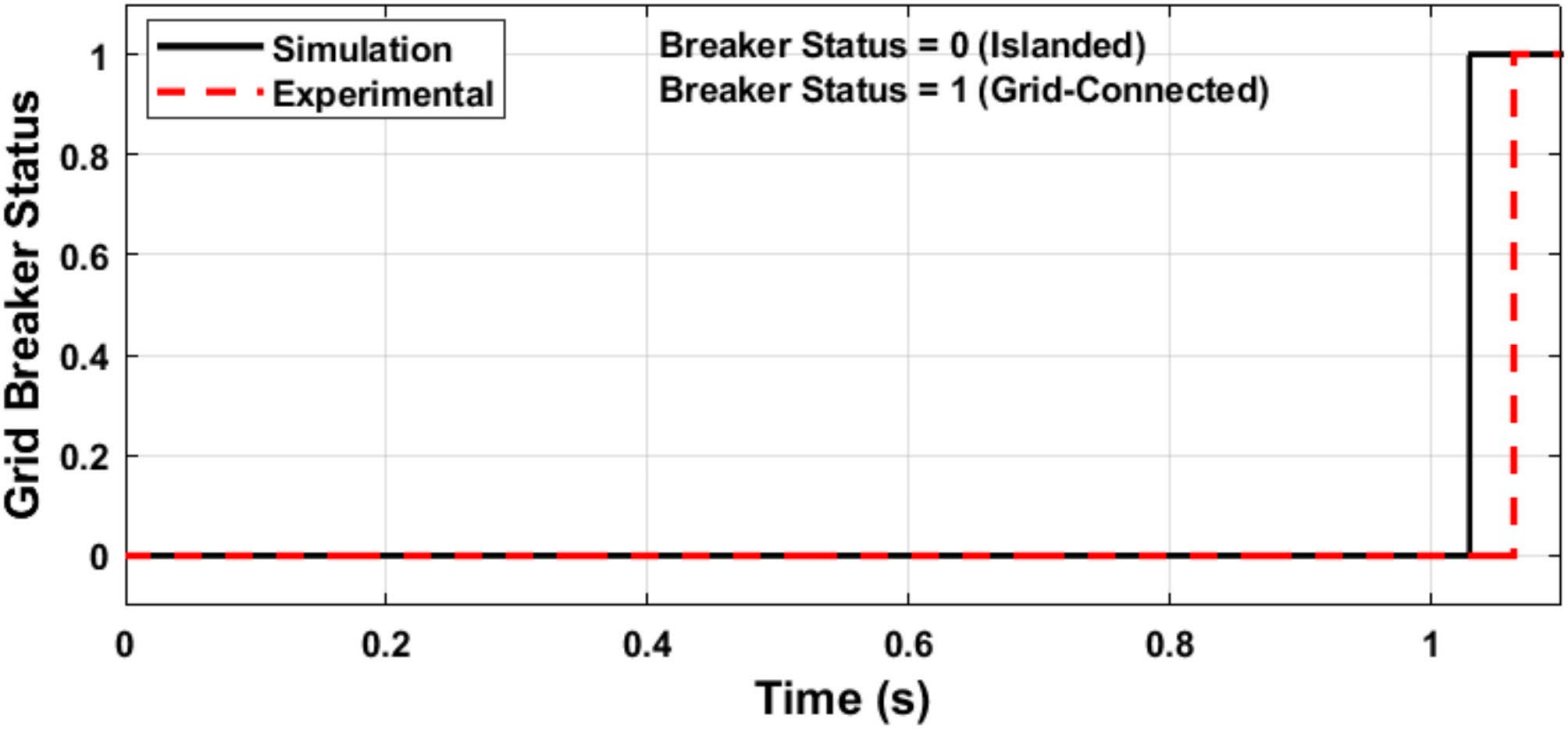
Status of grid circuit breaker.

The microgrid voltage amplitude and frequency are illustrated in Figs. 24 and 25, respectively. Figure 26 illustrates the status of the grid circuit breaker, highlighting the transfer from islanded mode to grid-connected mode following successful synchronization. The results exhibit minimal discrepancies between the simulation and experimental findings. This high level of consistency is attributed to the authors’ diligent optimization of key parameters, including sample time, baud rate, and data transfer rate, ensuring precise and reliable system performance.

## Conclusion

This study introduces an islanded microgrid system featuring one grid-feeding and two grid-forming distributed generators, modeled in the αβ stationary reference frame. Key control strategies include PR controllers for voltage and current regulation, droop control with virtual impedance for stability and power sharing, and restoration loops to address voltage and frequency deviations. A synchronization loop ensures seamless reconnection to the utility grid by minimizing voltage mismatches. The control parameters are optimized using the Electric Eel Foraging Optimization (EEFO) technique, which outperforms established methods such as PSO and GWO in terms of solution quality and convergence speed. The microgrid’s reliability and the performance of the proposed control system are validated under both steady-state and dynamic conditions through comprehensive simulations. Furthermore, experimental validation is carried out via hardware-in-the-loop (HIL) testing using the C2000 microcontroller LaunchPad XL TMS320F28379D kit, confirming the practical applicability and effectiveness of the proposed EEFO-based controllers.

## Future work

This research could be further extended by integrating renewable energy sources such as PV and FC systems, along with battery energy storage systems, to analyze the effects of generation fluctuations on the microgrid’s performance. Additionally, harmonic compensators could be incorporated into the voltage and current control loops to enhance the power quality of the microgrid when supplying nonlinear loads. Future work could also explore the implementation of a tertiary control level to optimize and regulate the power flow between the microgrid and the utility grid. Moreover, to enhance the efficiency of the EEFO algorithm regarding exploration and exploitation, hybridization with other optimization techniques could be considered.

## Data Availability

The authors would like to confirm that all data generated or analyzed during this study are included in this published article.

## Appendix

**Table:**

parameter	Value
Power circuit	
Nominal line-to-line voltage	380 V (RMS)
Nominal frequency	50 Hz
DC link voltage	800 V
Output inductance	5 mH
Output resistance	0.5 Ω
LC filter capacitance	10 $\:\mu\:$F
LC filter damping resistance	20 Ω
Feeder inductance	1 mH
Feeder resistance	65 m Ω
Grid resistance	65 m Ω
Grid inductance	1 mH
***Control systems***	
***Grid-Feeding control loops***	
Active power controller $$\:{k}_{pP}$$$$\:{k}_{iP}$$Reactive power controller $$\:{k}_{pQ}$$ $$\:{k}_{iQ}$$	0 0.5 6 15
***Grid-Forming control loops***	
***Primary control***	
Voltage PR controller $\:{k}_{pv}$ $\:{k}_{iv}$ $\:{\zeta\:}_{v}$	0.12 0.1 0.01
Current PR controller $\:{k}_{pi}$ $\:{k}_{ii}$ $\:{\zeta\:}_{i}$	13.6 228.5 0.102
Droop controller Cut-off frequency LPF $\:{m}_{P}$ $\:{m}_{pp}$ $\:{n}_{Q}$ $\:{L}_{V}$	9.425 rad/s 105 × 10^−6^ 8.4 × 10^−6^ 8.1 × 10^−4^ 1 mH
***Secondary control***	
Restoration controllers $\:{k}_{pvs}$ $\:{k}_{ivs}$ $\:{k}_{p\omega\:}$ $\:{K}_{i\omega\:}$ Synchronization controllers $\:{k}_{pvs}$ $\:{k}_{ivs}$ $\:{k}_{p\omega\:s}$ $\:{k}_{i\omega\:s}$	0.12 46.2 0 14.55 1 100 0.008 1.9

## Declarations

**Correspondence** and requests for materials should be addressed to M. A. Ebrahim.
